# Mechanistic and functional characterization of ABTB2 as a novel target for pancreatic cancer therapy

**DOI:** 10.1016/j.omton.2025.201080

**Published:** 2025-11-01

**Authors:** Nan Lyu, Olamide T. Olaoba, Qiongling Wang, Harinarayanan Janakiraman, Xinjian Liu, Ernest Ramsay Camp, Kun Cheng, Eric T. Kimchi, Yi Miao, Kevin F. Staveley-O’Carroll, Guangfu Li

**Affiliations:** 1 Department of Surgery, University of Missouri, Columbia, MO 65212, USA; 2 Pancreas Center, The First Affiliated Hospital of Nanjing Medical University, Nanjing, Jiangsu 210029, China; 3 Department of Surgery, University of Connecticut Health Center, Farmington, CT 06030, USA; 4 Department of Immunology, University of Connecticut Health Center, Farmington, CT 06030, USA; 5 Division of Surgical Oncology, Baylor College of Medicine, Houston, TX 77030, USA; 6 Division of Pharmacology and Pharmaceutical Sciences, School of Pharmacy, University of Missouri-Kansas City, Kansas City, MO, USA

**Keywords:** MT: Regular Issue, pancreatic ductal adenocarcinoma, PDAC, ankyrin repeat and BTB/POZ domain containing protein 2, ABTB2, TNF receptor associated protein 1, TRAP1, Wnt/β-catenin signaling pathway, targeted therapy

## Abstract

BTB/POZ domain-containing proteins are increasingly recognized for their context-dependent roles in cancer, acting as either tumor suppressors or oncogenic drivers depending on the cancer type. Among them, the function of ankyrin repeat and BTB domain-containing protein 2 (ABTB2) in pancreatic ductal adenocarcinoma (PDAC), a highly lethal malignancy, has remained unexplored. In this study, we employed comprehensive functional genomics approaches—siRNA/shRNA knockdown, CRISPR-Cas9 knockout, plasmid-based overexpression, and a Cre-LoxP transgenic mouse model—to systemically modulate *ABTB2* expression in human and murine PDAC cell lines, as well as in the KPC mouse model of PDAC. Our gain- and loss-of-function studies revealed that ABTB2 plays a pivotal tumor-suppressive role, significantly impairing PDAC cell oncogenicity *in vitro* and tumorigenesis *in vivo*. Importantly, therapeutic targeting of ABTB2 using adeno-associated virus serotype 2 (AAV2) and lipid nanoparticles (LNPs) demonstrated marked anti-tumor efficacy and synergized with 5-fluorouracil (5-FU) to enhance treatment outcomes. Transcriptomic analysis, immunoprecipitation, and functional assays demonstrated that ABTB2 interacts with tumour necrosis factor receptor-associated protein 1 (TRAP1), promoting its ubiquitin-dependent degradation and thereby suppressing key oncogenic Wnt/β-catenin and PI3K/Akt signaling pathways. Notably, TRAP1 inhibitors are currently in phase I clinical trials as potential anticancer agents. Our findings provide mechanistic insight and underscore the ABTB2/TRAP1 axis as a promising therapeutic target for PDAC treatment.

## Graphical abstract

**Figure:**
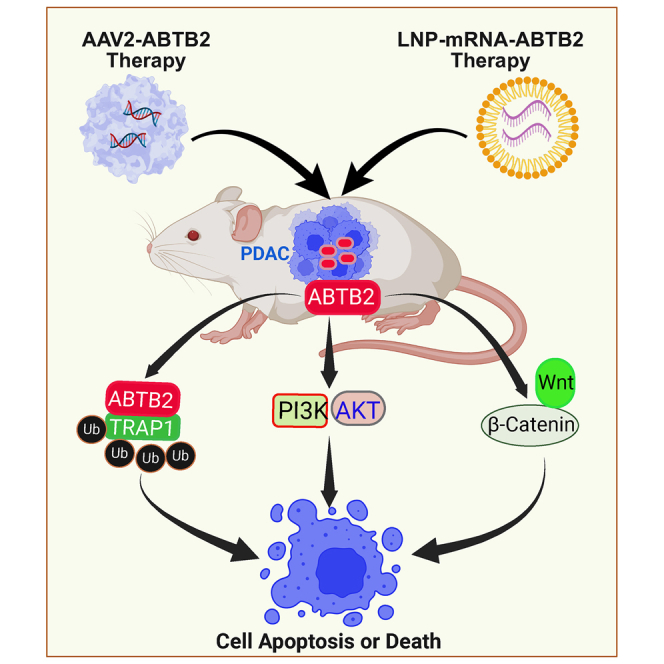

## Teaser

Nan and colleagues uncovered ABTB2 as a previously unrecognized but critically intrinsic suppressor of PDAC, demonstrating its powerful antioncogenic and antitumorigenic roles through diverse cellular functions and therapeutic strategies. Mechanistically, ABTB2 interacts with TRAP1 to trigger its degradation via ubiquitination and suppresses critical oncogenic pathways, including the Wnt/β-catenin and PI3/AKT pathways.

## Introduction

Pancreatic ductal adenocarcinoma (PDAC), the most prevalent form of pancreatic cancer, remains one of the deadliest malignancies worldwide. Although pancreatic cancer accounts for only 3% of all cancers, it is responsible for an alarming 7% of cancer-related deaths in the United States.^1^ According to projections by the American Cancer Society, pancreatic cancer is expected to become the second leading cause of cancer-related deaths by 2030.^1^^,^^2^ Globally, it causes over 200,000 deaths annually, with a 5-year survival rate of less than 6%.^3^^,^^4^^,^^5^ These sobering statistics reflect decades of stagnation in early detection, treatment efficacy, and long-term patient survival, underscoring the urgent need for new directions in pancreatic cancer research.^6^^,^^7^

Several factors contribute to the poor prognosis of PDAC, including aggressive metastasis, high rates of therapeutic resistance, frequent relapse, and limited surgical eligibility.^8^^,^^9^^,^^10^^,^^11^^,^^12^^,^^13^ Standard treatment options, including surgery, chemotherapy, and radiation therapy,^14^^,^^15^ offer only marginal benefits. Fewer than 20% of patients are candidates for surgical resection,^16^ and PDAC remains largely unresponsive to existing chemotherapeutics and radiation.^17^^,^^18^ Despite significant advances in immunotherapy, approaches such as immune checkpoint inhibitors and chimeric antigen receptor (CAR)-T cells have shown little to no efficacy in PDAC,^19^^,^^20^ highlighting the urgent need for novel, effective therapeutic strategies.

Targeted molecular therapies have shown significant promise for cancer treatment and are currently being explored for various cancers,^21^^,^^22^^,^^23^^,^^24^^,^^25^^,^^26^ yet similar progress has not been realized in PDAC. Clinical trials targeting known oncogenic drivers have yielded disappointing results,^27^ and median patient survival remains below 1 year.^28^ This highlights a critical need to identify and validate new molecular targets that could drive more effective and specific treatments for PDAC.

BTB/POZ domain-containing proteins are emerging as promising targets for cancer therapy due to their roles in transcriptional regulation, ubiquitination, and oncogenic signaling pathways.^29^^,^^30^^,^^31^^,^^32^^,^^33^^,^^34^^,^^35^^,^^36^ Among them, ABTB2 has gained attention for its involvement in PTEN-dependent tumor suppression in ovarian tumors and its association with drug resistance in breast and thyroid cancers,^37^^,^^38^ highlighting its relevance across multiple cancer contexts. Despite this, ABTB2 remains entirely uncharacterized in PDAC, one of the most treatment-refractory^39^^,^^40^ and molecularly distinct cancers. This represents a significant knowledge gap in PDAC reserach. Given its potential functions in protein complex assembly, transcriptional repression, and post-translational modification, ABTB2 may be a key regulator of oncogenic signaling pathways that drive PDAC progression. This convergence of its mechanistic relevance, cancer-associated domain structure, and the complete absence of PDAC-specific investigation strongly promoted us to explore ABTB2 as a novel and potentially impactful therapeutic target in PDAC.

In this study, we utilized a comprehensive suite of functional genomics tools, including siRNA, shRNA, CRISPR-Cas9, and expressing plasmids, to manipulate *ABTB2* expression in PDAC cells. We established and characterized stable PDAC cell lines with *ABTB2* knockdown (KD), knockout (KO), or overexpression (OE) to determine its role in tumorigenesis. Our *in vitro* and *in vivo* findings consistently demonstrate that *ABTB2* suppression promotes PDAC cell proliferation, migration, and survival, whereas ABTB2 OE inhibits these oncogenic traits. These effects were validated in multiple human and murine PDAC models, including orthotopic, transgenic, and patient-derived xenograft (PDX) systems.

To translate these findings into a therapeutic strategy, we developed a recombinant adeno-associated virus serotype 2 (AAV2) vector expressing *ABTB2* under the control of a cyclooxygenase-2 (COX2) promoter, enabling tumor-selective expression. Systemic administration of this vector markedly suppressed tumor growth in multiple PDAC models without eliciting detectable toxicity. Furthermore, encapsulating ABTB2 mRNA in lipid nanoparticles (LNPs) yielded potent antitumor activity, which synergized with 5-fluorouracil (5-FU) to further enhance therapeutic efficacy. Mechanistically, we uncovered a novel regulatory axis in PDAC by identifying a previously unrecognized interaction between ABTB2 and the mitochondrial chaperone tumour necrosis factor receptor-associated protein 1 (TRAP1), using immunoprecipitation coupled with mass spectrometry. This interaction promotes TRAP1 degradation, ultimately disrupting key oncogenic signaling pathways, including Wnt/β-catenin and Phosphatidylinositol 3-kinase / v-Akt Murine Thymoma Viral Oncogene (PI3K/Akt). Notably, TRAP1 has been independently recognized as an oncogenic driver, with several targeted inhibitors currently undergoing preclinical and clinical evaluation (NCT04827810).^41^^,^^42^^,^^43^ Our findings not only highlight ABTB2 as a promising molecular target in PDAC but also shed light on a novel tumor-suppressive mechanism mediated by TRAP1. Collectively, our work provides a strong preclinical rationale for translating ABTB2-based therapies into future clinical trials for this intractable malignancy.

## Results

### ABTB2 dysregulation significantly impacts PDAC cell oncogenicity *in vitro*

**Figure 1:**
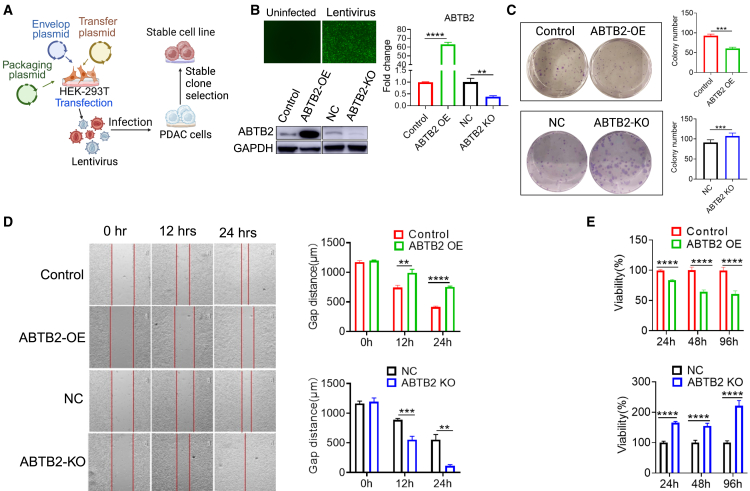
Gain- and loss-of-function experiments demonstrate the crucial role of ABTB2 in PDAC development *in vitro* (A) Establishment of stable Panc02 cells with *ABTB2* knockout (KO) or overexpression (OE). A schematic illustrates the process of creating stable Panc02 cells with either ABTB2-OE or ABTB2-KO. Plasmids carrying ABTB2 or gRNA/Cas9 were co-transfected into HEK-293T cells with helper vectors psPAX2 and pMD2G to package lentivirus particles. Post overnight culture, the supernatant containing lentiviral particles was used to infect Panc02 cells. After 72 h, cells were subcultured at low density into 96 well-plates. Monoclonal cells that stably express *ABTB2* or gRNA/Cas9 were selected using antibiotics for subsequent expansion. (B) Validation of ABTB2 KO and OE. The transduction efficiency of recombinant lentivirus in Panc02 cells was monitored by GFP expression under a microscope (upper left). ABTB2 KO or OE at the mRNA and protein levels was confirmed by RT-qPCR (right) and western blotting (lower left). (C) Impact of ABTB2 KO and OE on Panc02 cell colony formation. Panc02 cells with ABTB2-OE or ABTB2-KO were seeded into 6-well plates at a density of 200 cells per well and cultured for 7 or 10 days, then the cells were fixed with 6% v/v glutaraldehyde and stained with 0.5% w/v crystal violet. The colonies of Panc02 cells with ABTB2-OE (upper left) and Panc02-KO (lower left) were imaged and counted. (D) Impact of ABTB2 KO and OE on Panc02 cell migration: Panc02 cells with ABTB2-OE or ABTB2-KO were seeded into 24-well plate with a wound healing insert which was removed the second day. The wound closure was imaged under microscopy for assessing cell migration with ABTB2 OE (upper left) and ABTB2 KO (lower left); the accumulated gap diameters were counted (right). (E) Impact of ABTB2 KO and OE on Panc02 cell viability. Panc02 cells with ABTB2-OE or ABTB2-KO were seeded and cultured in 96-well plate. 16 h later, 10 μL of MTT labeling reagent was added to each well and incubated for 3 h, after which the absorbance of purple formazan crystals was measured. Cell viability with ABTB2-OE (left) and ABTB2-KO (right) was calculated as a percentage relative to the respective control. All cell culture experiments were conducted in at least three replicates (*n* = 3). Statistical significance is denoted as ∗*p* < 0.05, ∗∗*p* < 0.01, and ∗∗∗*p* < 0.001.

There is a lack of understanding regarding the role of ABTB2 in human PDAC. We first investigated the relationship between ABTB2 and human PDAC. Immunohistochemistry (IHC) detected a markedly decreased *ABTB2* expression in human PDAC tumors compared to peritumor tissues (Figure S1A). This decrease was also observed in mouse PDAC tumors in KPC mice relative to the normal pancreas in wild-type mice (Figure S1B). These results suggest a negative correlation between *ABTB2* expression and PDAC growth. To further determine the exact role of ABTB2 in PDAC, functional genomics strategies were used to induce ABTB2 OE and KO in mouse Panc02 cells. Specifically, as depicted in Figure 1A, the transduction of lentiviruses expressing *ABTB2* or gRNA/Cas9, followed by antibiotic selection, was utilized to establish stable Panc02 cells with ectopic ABTB2 OE (ABTB2-OE) or ABTB2 KO (ABTB2-KO). Western blotting (Figure 1B, lower left) and qPCR (Figure 1B, right) validated ABTB2 OE or depletion at both the protein and mRNA levels. The clonogenic assay showed that Panc02 cells with ABTB2 OE exhibited reduced colony formation, while ABTB2-KO resulted in increased colony formation compared to their respective controls (Figure 1C). The wound-healing assay demonstrated that ABTB2 OE significantly suppressed Panc02 cell migration, whereas ABTB2-KO markedly promoted cell movement (Figure 1D). A microplate reader assay demonstrated a decrease in Panc02 cell viability with ABTB2 OE and an increase with ABTB2-KO (Figure 1E). These effects were further verified in mouse UN-KPC-961 cells and human Panc-1 cells (Figure S2). Collectively, these results suggest that ABTB2 significantly influences the oncogenicity of both human and mouse PDAC by exerting multiple suppressive effects on PDAC proliferation, migration, and viability *in vitro*.

### ABTB2 dysregulation significantly impacts PDAC tumorigenicity *in vivo*

**Figure 2:**
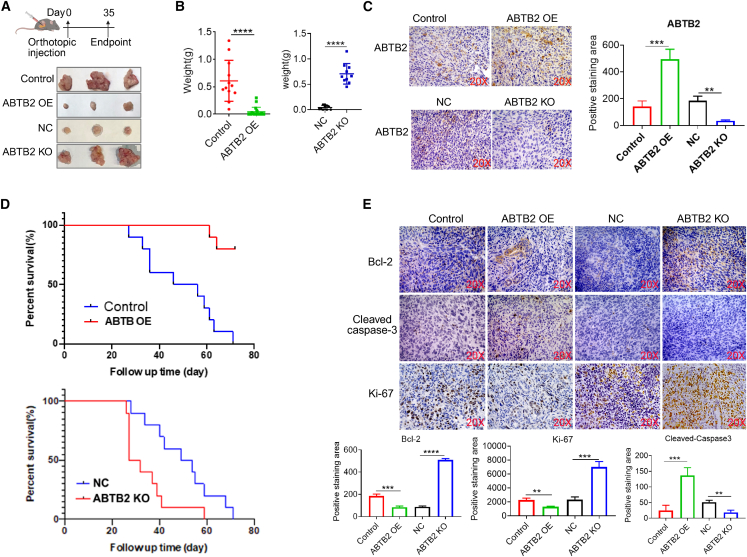
Impact of ABTB2 ectopic expression or depletion on PDAC growth *in vivo* (A) Establishment of orthotopic PDAC-bearing mice: A schematic illustrates the preparation of orthotopic PDAC and endpoint euthanasia. Wild-type C57BL/6 mice received intra-pancreatic injections of Panc02 cells with ABTB2-OE (5 × 10^4^ per mouse) or ABTB2-KO (2.5 × 10^4^ per mouse), with corresponding vehicle as controls. Post 35 days, mice were euthanized, and tumors were harvested for further analysis. (B) Tumor size and weight measurement: Macroscopic photographs of tumors from each mouse are shown on the left, with cumulative tumor weights displayed on the right. The date indicated an inverse relationship between *ABTB2* expression and tumor growth. (C) Immunohistochemistry (IHC) for detection of *ABTB2* expression: IHC was performed to validate *ABTB2* expression levels in the tumors. Tumors induced by Panc02 cells with ABTB2-OE or ABTB2-KO, along with their corresponding vehicles, were processed, sectioned, and stained with an ABTB2 antibody to assess *ABTB2* expression levels. (D) Kaplan-Meier survival analysis of tumor-bearing mice: viable mice with tumors induced by ABTB2-OE or ABTB2-KO cells were documented daily. Survival rates in each group were calculated over time to construct the Kaplan-Meier Curve. (E) IHC for detection of cell growth and apoptosis markers: Tumors induced with ABTB2-OE or ABTB2-KO cells were processed, sectioned, and stained with antibodies against Bcl2, ki67, and cleaved capase-3. The area of positive staining was quantified using ImageJ software. (*n* = 10). Statistical significance is indicated by ∗*p* < 0.05, ∗∗*p* < 0.01, and ∗∗∗*p* < 0.001.

Given ABTB2’s critical role in PDAC cellular events *in vitro*, we explored its impact as an intrinsic factor on PDAC tumor growth *in vivo*. As depicted in Figure 2A, we initiated orthotopic PDAC tumors in wild-type C57BL/6 mice through intra-pancreatic injection of stable Panc02 cells with ABTB2 OE or KO. Upon reaching the 35-day mark post cell inoculation, all mice were euthanized. Macroscopic examination revealed a significantly reduced tumor size derived from ABTB2-OE Panc02 cells, while an increase in tumor size was observed in tumors induced by ABTB2-KO cells, relative to their respective control groups (Figure 2B, left). These findings were further validated by the quantified tumor weights (Figure 2B, right). These observed effects were also corroborated in mouse UN-KPC-961 and human Panc-1 cells (Figure S3). Kaplan-Meier survival analysis demonstrated a marked extension of lifespan in mice receiving Panc02 cells with ABTB2-OE, whereas a decrease in survival time was noted in mice receiving Panc02 cells with ABTB2-KO, compared to their respective controls (Figure 2C). IHC analysis identified reduced Bcl-2 and Ki-67 expression, alongside elevated cleaved caspase-3 expression, in tumors derived from ABTB2-OE Panc02 cells. In contrast, tumors induced by ABTB2-KO cells exhibited the opposite patten (Figure 2D). Using a Cre-LoxP-based genetic strategy, we established spontaneous PDAC models—AKPPC and KPPC—by interbreeding mice carrying homozygous Trp53^R172H^ mutations (PP), pancreas-specific Kras^G12D^ and p48-Cre (KC), and a heterozygous ABTB2 transgene (A). Phenotypic analysis and tumor burden assessment revealed that ABTB2 significantly suppressed spontaneous PDAC progression and prolonged survival in mice (Figure S4). Collectively, our findings indicate that gain and loss of ABTB2 function significantly affect PDAC tumor development and recipient mice survival, involving modulation of tumor cell viability and apoptosis. These results underscore the pivotal role of ABTB2 in suppressing PDAC progression.

### ABTB2-encoding AAV2 therapeutically suppresses mouse PDAC tumor growth

**Figure 3:**
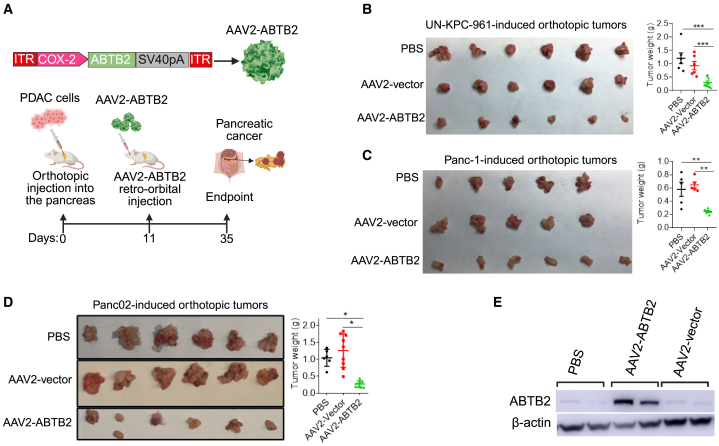
ABTB2-recombinant AAV2 therapeutically suppresses orthotopic PDAC tumor growth in mice (A) Schematic diagrams depicting the preparation of ABTB2-recombinant AAV2 (AAV2-ABTB2) and outlining the administration protocol. Recombinant AAV2-ABTB2 was prepared through cDNA subcloning, amplification, CsCl ultracentrifugation, and qPCR titration, achieving a final concentration of 2 × 10^13^ vg/ml. Orthotopic PDAC-bearing mice were established in wild-type C57BL/6 mice by intra-pancreatic injection of the indicated mouse PDAC cells at 5 × 10^4^ cells/mouse. Subsequently, the mice were randomly divided into three groups and received injections of recombinant AAV2-ABTB2 at 10^11^ vg/mouse. AAV2 vector and PBS were used as controls. All mice were euthanized on day 35 post-cell inoculation. (B to D) Recombinant AAV2-ABTB2 injection inhibits the growth of PDAC tumors induced by mouse UN-KPC-961 cells (B), human Panc-1 cells (C), and mouse Panc02 cells (D). On day 35 post-cell inoculation, all mice were euthanized. Macroscopic tumors along with their corresponding weights for each mouse were meticulously recorded. (E) Recombinant AAV2-ABTB2 treatment leads to enhanced *ABTB2* expression. Western blotting was used to assess *ABTB2* expression levels in Panc02-induced tumors with or without AAV2-ABTB2 treatment. (*n* = 6). Statistical significance is indicated by ∗*p* < 0.05, ∗∗*p* < 0.01, and ∗∗∗*p* < 0.001.

*In vitro* and *in vivo* studies have identified ABTB2 as a critical factor suppressing PDAC progression, which prompted us to investigate its potential as a target for developing anti-PDAC therapy. AAV2 is a popular vector in gene therapy due to its low immunogenicity and lack of pathogenicity.^44^ It has been used as a vector to treat various human diseases, including hemophilia, spinal muscular atrophy, and inherited retinal diseases.^45^^,^^46^^,^^47^ Thus, we constructed an ABTB2 recombinant virus using AAV2 as the vector under the control of the COX2 promoter, named AAV2-ABTB2 (Figure 3A, upper). COX2 is not pancreas-specific but is notably upregulated in human pancreatic cancer.^48^^,^^49^ In an orthotopic PDAC tumor model induced by mouse UN-KPC-961 cells, mice received an intravenous (i.v.) injection of AAV2-ABTB2 at a dose of 10^11^ GC/mouse on day 11 post cell inoculation (Figure 3B, upper). Macroscopic examination showed a markedly reduced tumor size in AAV2-ABTB2-treated mice compared to those with PBS or AAV2 control virus (Figure 3B). Correspondingly, tumor measurements showed a reduced tumor weight in AAV2-ABTB2-treated mice (Figure 3B). The therapeutic effect of recombinant AAV2-ABTB2 was further validated in tumors induced by human Panc-1 cells (Figure 3C) and mouse Panc02 cells (Figure 3D). Western blotting confirmed that AAV2-ABTB2 treatment significantly enhanced the production of ABTB2 in tumors relative to treatment with PBS or AAV2 vector virus (Figure 3E). Toxicity assays indicated that treatment with AAV2-ABTB2 did not significantly impact liver function, glomerular filtrate rate, cardiac function, or mean corpuscular volume; additionally, other hematological components remained unchanged, suggesting no detectable side effects induced by AAV2-ABTB2 (Figure S5). These findings indicate that recombinant ABTB2 serves as a monotherapy capable of therapeutically suppressing mouse tumor growth induced by distinct types of PDAC cells, an effect attributed to increased production of ABTB2 within tumors.

### ABTB2-encoding AAV2 therapeutically suppresses human PDAC tumor growth

**Figure 4:**
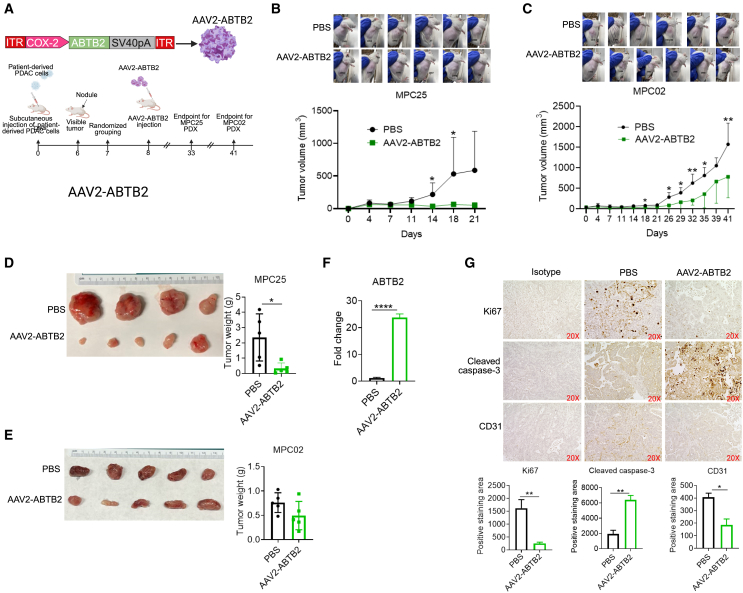
ABTB2-recombinant AAV2 inhibits human PDAC tumor growth in a patient-derived xenograft model (A) Schematic diagram illustrates ABTB2-recombinant AAV2 preparation (what difference with Figure 3 or overlap), PDX model establishment, and AAV2-ABTB2 treatment. The ABTB2-recombinant virus was engineered using AAV2, as depicted in Figure 3A. For the creation of PDX mice, each NSG mouse received a subcutaneous injection of human PDAC tumor cells from two patients (MPC02 or MPC25) at 1 ×10^6^ cells per mouse. The resulting tumors were serially transplanted into subsequent NSG mice. In this study, fourth-generation PDX mice harboring human patient MPC02 tumors (4PDX-MPCO2) and fifth-generation PDX mice with human tumors derived from patient MPC25 (5PDX-MPC25) were treated with AAV2-ABTB2 on day 8. PBS was used as the control. 4PDX-MPC02 mice were euthanized on day 41 and 5PDX-MPC25 mice on day 33. (B and C) AAV2-ABTB2 treatment led to a reduction in tumor volume. The sizes of human MPC02 (B) and MPC25 tumors (C) throughout the duration of the experiment were measured at the indicated intervals using a Vernier Caliper. The results were graphically represented to depict changes in tumor volume. (D and E) Recombinant AAV2-ABTB2 treatment led to the reduction in tumor weight. On day 33, mice in each group were euthanized to harvest tumors. Tumor weights from both 4PDX-MPCO2 (D) and 5PDX-MPC25 (E) mice were decreased following treatments. (F) AAV2-ABTB2 treatment increased mRNA expression of ABTB2 in PDX tumors. RNA was isolated from tumors of treated and untreated mice, and ABTB2 mRNA expression was determined by RT-qPCR. (G) Recombinant AAV2-ABTB2 treatment inhibited tumor growth, induced apoptosis, and suppressed vascularization. A portion of tumors was sectioned for IHC. Results indicated that AAV2-ABTB2 reduced Ki67 and CD31 expression and increased cleaved caspase-3 production. *n* = 6. Significance levels are denoted as ∗*p* < 0.05, ∗∗*p* < 0.01, and ∗∗∗*p* < 0.001.

After demonstrating the effectiveness of AAV2-ABTB2 in mouse PDAC tumors, we further evaluated its therapeutic potential in suppressing human PDAC tumors. To this end, we developed a PDX model in immunodeficient NOD.Cg-*Prkdc^scid^ Il2rg^tm1Wjl^*/SzJ (NSG) mice by subcutaneously injecting single-tumor-cell suspension from two patients with PDAC (MPC02 and MPC25). Upon the appearance of visible nodules on day 9, the mice were randomly assigned to receive an intratumoral injection of AAV2-ABTB2 at a dose of 1 × 10^11^ vg per mouse or control PBS. The intratumoral injection method was selected because tumors in the subcutaneous mouse model can be directly accessed. Tumor growth was consecutively monitored by measuring tumor size until day 33 (Figure 4A). In comparison with PBS, AAV2-ABTB2 therapy significantly reduced tumor volume in tumors derived from MPC25 by day 11 and from MPC02 by day 18, with a sustained effect until the endpoint of the experiment on day 33, demonstrating the durability of AAV2-ABTB2 therapy (Figures 4B and 4C). This therapeutic suppression was further quantified by decreased tumor weights at the endpoint (Figures 4D and 4E). RT-qPCR confirms that AAV2-ABTB2 treatment significantly enhanced *ABTB2* expression in the PDX tumors (Figure 4F). IHC analysis identified reduced Ki-67 and CD31 expression levels, along with increased cleaved caspase-3 levels (Figure 4G). These findings were also validated at the mRNA level by qPCR. Overall, recombinant ABTB2 exerts therapeutic effect on both mouse and human PDAC tumors by suppressing tumor cell proliferation, inhibiting vascularization, and promoting tumor apoptosis, highlighting its potential clinical application for the treatment of human PDAC.

### LNP-encapsulating ABTB2 mRNA synergizes with 5-FU to suppress PDAC

**Figure 5:**
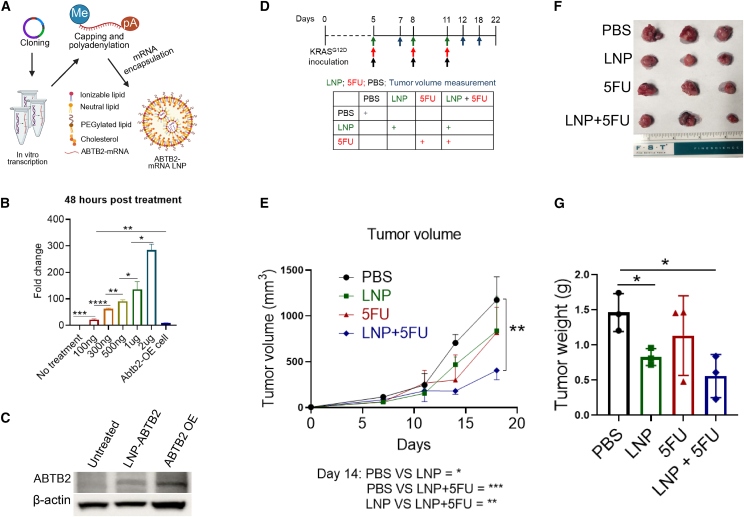
LNP-mRNA-ABTB2 in combination with 5-FU induced synergistic therapeutic suppression of PDAC tumors (A) Construction of LNP-encapsulated ABTB2-mRNA. Schematics show ABTB2-mRNAs encapsulated in LNPs, named LNP-mRNA-ABTB2. (B) Transduction efficacy of LNP-mRNA-ABTB2 in KRAS^G12D^ cell. KRAS^G12D^ cells was seeded into 6-well plates at a dose of 5 × 10^5^/well and incubated at 37°C with 5% CO_2_ and saturated humidity. After overnight culture, LNP-mRNA-ABTB2 was added to each well at varying doses from 100 ng to 2 μg. after 48 h, cells were harvested to isolate total RNA, which was reversed transcribed into cDNA to evaluate ABTB2 mRNA expression levels by RT-qPCR. Stable KRAS^G12D^ cells with ABTB2-OE were used as a positive control. (C) LNP-mRNA-ABTB2 transduction led to ABTB2 production. As described in (B), KRAS^G12D^ cells transduced with LNP-mRNA-ABTB2 for 48 h showed increased ABTB2 production, which was detected by western blotting. (D) LNP-mRNA-ABTB2 treatment design. KRAS^G12D^ cells suspended in 15% Matrigel was subcutaneously injected into the left flank of 8-week-old C57BL/6 mice. From day 5, mice received injections of 5 μg LNP-mRNA-ABTB2 and 25 mg/Kg 5-FU every 3 days for three treatments. Mice receiving monotherapy with LNP, 5FU, or PBS were used as controls. (E) LNP-mRNA-ABTB2 and 5-FU alone led to a reduction in tumor volumes with a great effect seen in their combination. From the first treatment, the longest (L) and shortest (W) diameters of the subcutaneous implants were regularly measured. Tumor volume was calculated using the formula: L/2 × (W^2^). (F and G). LNP-mRNA-ABTB2 and 5-FU alone led to a reduction in tumor weights, with an increased effect seen in their combination. (G) Twenty-two days after cell inoculation, tumors were isolated from mice receiving the indicated treatments, and tumor weights were measured. All cell culture experiments were conducted in at least four replicates (*n* = 4), and animal experiment in three replicates (*n* = 3). Statistical significance is denoted as ∗*p* < 0.05, ∗∗*p* < 0.01, and ∗∗∗*p* < 0.001.

In comparison with AAV as a vector, LNPs offer many advantages in mRNA-based therapy, such as delivering mRNA to the cytoplasm—the site of mRNA-mediated protein synthesis—accommodating larger mRNA payloads (up to 10–15 kb), enabling scalable production, and exhibiting low immunogenicity.^50^^,^^51^^,^^52^^,^^53^ These features make LNPs especially suitable for developing ABTB2-based therapy for human PDAC. Employing LNPs as vehicles to encapsulate ABTB2 mRNA, we produced LNP-loaded ABTB2 mRNAs, termed LNP-mRNA-ABTB2 (Figure 5A). Dose-response analysis revealed that transducing cells in a six-well plate with LNP-mRNA-ABTB2 at 100 ng/well produced significantly higher ABTB2 mRNA levels compared with stable KRAS^G12D^ cells with ABTB2-OE (Figure 5B), indicating the high efficiency of LNP-mRNA-ABTB2 transduction. Western blotting showed that ABTB2-mRNA-LNP transduced cells produced ABTB2 protein at levels equivalent to those in stable ABTB2-OE KRAS^G12D^ cells (Figure 5C). We tested the therapeutic potential of LNP-mRNA-ABTB2 on KRAS^G12D^ cell-induced subcutaneous tumors in wild-type mice, administering every 3 days for a total of three doses (Figure 5D). Significant tumor growth inhibition was observed with both LNP-mRNA-ABTB2 and 5-FU monotherapies; notably, their combination produced a synergized therapeutic outcome (Figure 5E). We choose to initially investigate the therapeutic potential of ABTB2 in combination with 5-FU because 5-FU is not only a key component of FOLFIRINOX, a current standard of care, but is also widely used in chemoradiation protocols. Quantification of tumor weights at the end of the experiments further validated these findings (Figures 5F and 5G). These results suggest that LNP-mRNA-ABTB2, like AAV2-ABTB2, exerts therapeutic suppression on PDAC and produces a synergistic effect with 5-FU, representing a new and powerful therapeutic strategy.

### Dysregulated expression of ABTB2 modulates the Wnt/β-catenin signaling pathway

**Figure 6:**
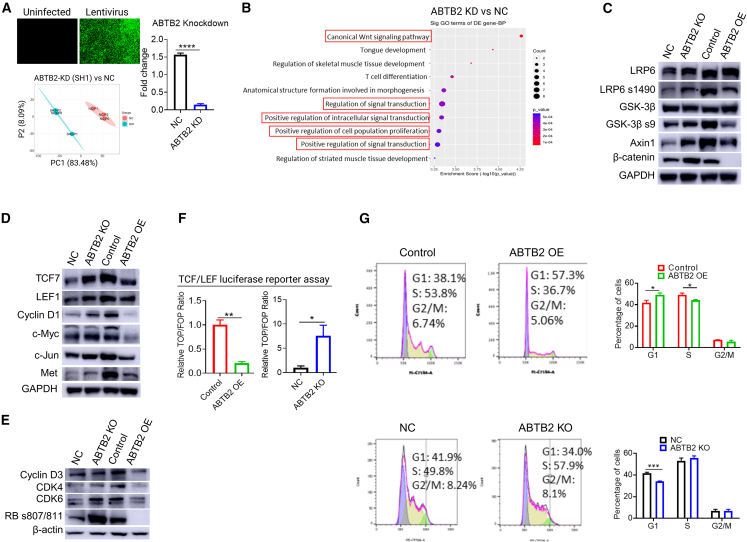
ABTB2 modulates the canonical Wnt/β-catenin signaling pathway (A) ABTB2 KD induces transcriptomic profile alterations in KRAS^G12D^ cells. GFP expression indicated the efficacy of ABTB2-shRNA lentivirus transduction (upper right). qPCR validated ABTB2 knockdown induced by ABTB2-shRNA-lentivirus (right). Principal component analysis revealed a clear distinction between the transcriptomes of ABTB2-KD KRAS^G12D^ cells and control cells (lower right). (B) Gene Ontology (GO) analysis identified the enriched biological processes induced by ABTB2-KD in KRAS^G12D^ cells. Fisher’s exact test identified the overlaps, differentially expressed genes, and the GO annotation list, with canonical Wnt signaling ranked as the top term in ABTB2-KD KRAS^G12D^ cells. (C) Western blotting demonstrated ABTB2’s regulatory effect on transmembrane and cytosolic Wnt/β-catenin signaling molecules. ABTB2 OE reduced the expression of LRP6 s1490, GSK3β s9, β-catenin, and Axin, whereas ABTB2 KO increased the expression of these molecules. GAPDH was used as a loading control. (D) Western blotting indicated that ABTB2 regulates nuclear Wnt/βcatenin signaling molecules. ABTB2-OE inhibited the expression of nuclear TCF7, LEF1, c-Myc, c-Jun, Met, and cyclinD1, whereas ABTB2-KO increased the expression of these molecules. (E) Western blotting revealed that ABTB2 modulated the expression of downstream Wnt/β-catenin signaling molecules. ABTB2-OE inhibited the expression of downstream Wnt/β-catenin signaling molecules, including cyclin D3, CDK4, CDK6, and phosphorylated retinoblastoma protein (RB s807/811), whereas the ABTB2-KO increased their expression. Actin was used as a loading control. (F) TCF/LEF luciferase reporter assay demonstrated ABTB2’s effect on Wnt/β-catenin pathway activity. ABTB2-OE reduced relative luciferase units (RLU), with reporter luciferase activity normalized to control luciferase activity (Renilla), whereas ABTB2 KO increased RLU. (G) Flow cytometry revealed ABTB2’s function in cell cycle regulation. ABTB2-OE induced cell cycle arrest in the G1 and S phases, whereas ABTB2-KO induced cell arrest primarily in the G1 phase.

Uncovering the signaling pathways and identifying key factors responsible for ABTB2-induced PDAC suppression will not only significantly enhance our understanding of ABTB2 but also provide new targets for better PDAC management. Therefore, we proposed to explore the functional mechanisms of ABTB2 in PDAC with a loss-of-function approach. In this regard, we used lentivirus expressing shRNA to knock down ABTB2 in KRAS^G12D^ cells, followed by RNA sequencing. The data were analyzed using principal-component analysis (PCA), a widely used statistical method for identifying patterns and relationships within data. The results showed clear distinctions in gene expression profiles between ABTB2-KD cells and control cells (Figure 6A). By categorizing genes into hierarchical groups based on their roles using Gene Ontology (GO) analysis^31^ and determining nonrandom associations with ABTB2 KD via Fisher’s exact test,^32^ we found that Wnt signaling emerged as the highest-ranked pathway (Figure 6B), known for its critical role in regulating cancer progression and development. Western blotting detected reduced protein expression of LRP6 s1490, GSK3β s9, β-catenin, and Axin in ABTB2-OE cells compared with control cells. Conversely, ABTB2-KO cells exhibited the opposite expression pattern (Figure 6C). IHC confirmed the regulatory effect of ABTB2 dysregulation on β-catenin production in PDAC tumors (Figure S6). All of these factors belong to the Wnt/β-catenin signaling pathway family: LRP6 is a co-receptor of Wnt protein, while GSK3β and Axin are components of the destruction complex that regulates β-catenin levels.^54^ Furthermore, western blotting detected decreased expression of TCF7, LEF1, cyclinD1, c-Myc, c-Jun, and Met in ABTB2-OE cells, but increased expression in ABTB2-KO cells (Figure 6D). TCF7 and LEF1 are transcription factors that interact with β-catenin to activate the transcription of cyclin D1, c-Myc, and c-Jun, thereby modulating cell proliferation and survival. Met is a receptor that can be regulated by Wnt signaling, contributing to cancer progression and metastasis. These results strongly suggest that the Wnt/β-catenin pathway is implicated in ABTB2-induced suppression of pancreatic cancer.

Our RNA-Seq data uncovered the enrichment of Wnt/β-catenin signaling in association with ABTB2 dysregulation, and previous studies have demonstrated that aberrant activation of Wnt/β-catenin signaling is tightly linked to cancer development and progression through the abnormal expression of cyclin D1/D3, CDK4, and CDK6.^55^^,^^56^^,^^57^^,^^58^ These proteins form a complex that phosphorylates and inactivates the tumor suppressor RB;^59^ therefore, we further examined the expression of these proteins in ABTB2-OE and KO cells. Western blotting detected reduced production of cyclin D1/D3, CDK4, and CDK6 cells in ABTB2-OE cells, but increased expression of these factors in ABTB2-KO cells (Figure 6E). The regulatory effect of ABTB2 on these factors were also verified by qPCR at the mRNA level by increasing or decreasing ABTB2 production (Figure S7). Using a TCF/LEF luciferase reporter assay, a powerful tool for studying the activity of the Wnt/β-catenin signaling pathway, we detected decreased luciferase activity in ABTB2-OE cells but increased activity in ABTB2-KO cells, confirming that ABTB2 suppresses Wnt/β-catenin activity (Figure 6F). Cyclin D1/D3, CDK4, and CDK6 play crucial roles in regulating the cell cycle. Using flow cytometry with propidium iodide (PI) staining, we observed that ABTB2-OE, but not ABTB2-KO, induced arrest of the cell cycle in the G1 phase (Figure 6G). These results suggest that ABTB2 suppresses PDAC via the Wnt/β-catenin signaling pathway associated with cell cycle regulation.

### ABTB2 dysregulation disrupts PDAC growth and apoptosis signaling pathways

**Figure 7:**
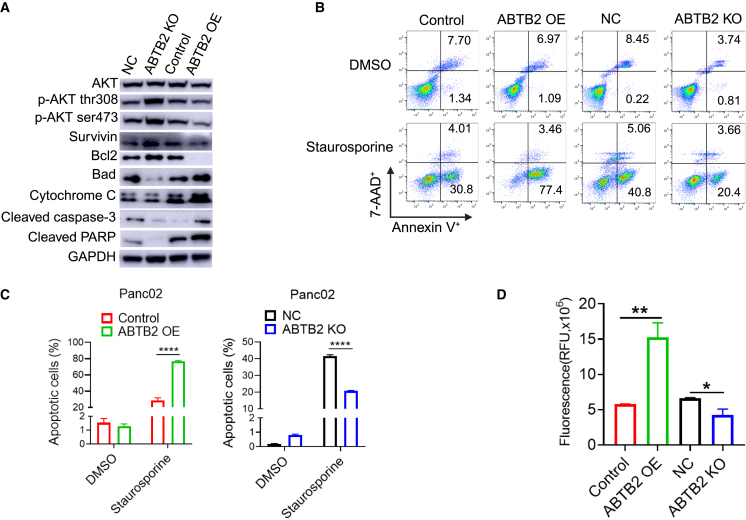
ABTB2 regulates cell death and survival signaling pathways (A) Western blotting confirmed the impact of ABTB2 on apoptotic and survival factors. ABTB2-OE inhibited the expression of Akt, pAkt (Thr308), pAkt (Ser473), survivin, and BCl2 in Panc02 cells, but promoted the expression of Bad, cytochrome *c*, cleaved caspase-3, and cleaved PARP, whereas ABTB2-KO produced the opposite effects. GAPDH was used as a loading control. (B) Representative flow cytometry showing the effect of ABTB2 on cell apoptosis. The frequency of staurosporine-induced cell death increased in ABTB2-OE Panc02 cells but decreased in ABTB2-KO Panc02 cells. (C) Cumulative summary of data shown in (B). (D) Caspase-Glo 3/7 luciferase assay demonstrating the effect of ABTB2 on caspase-3/7 activity. Increased and decreased caspase-3/7 activities were detected in ABTB2-OE and ABTB2-KO Panc02 cells, respectively. All cell culture experiments were conducted in at least three replicates (*n* = 3). Statistical significance is denoted as ∗*p* < 0.05, ∗∗*p* < 0.01, and ∗∗∗*p* < 0.001.

Tumor suppression is critically involved in the regulation of cell growth and apoptosis. Western blotting revealed a markedly reduced expression of antiapoptotic factors—phosphorylated Akt (thr308 and ser473), survivin, and BCL-2—in ABTB2-OE cells, accompanied by an increased expression of proapoptotic factors—Bad, cytochrome *c*, cleaved caspase-3, and cleaved PARP—in ABTB2-OE cells (Figure 7A). The opposite patterns of these growth and apoptotic factors were observed in ABTB2-KO cells (Figure 7A), suggesting that ABTB2 induces PDAC suppression by regulating cell growth and apoptosis. Next, we investigated whether ABTB2 OE or KO could promote or attenuate staurosporine-induced cell apoptosis. Using flow cytometry with 7-AAD and Annexin V staining, we detected increased frequencies of both Annexin V^+^7-AAD^−^ cells (early apoptotic cells) and Annexin V^+^7-AAD^+^cells (late apoptotic cells) in ABTB2-OE cells, but reduced frequencies in ABTB2-KO cells (Figure 7B). Accumulated results demonstrated the reliability of this finding (Figure 7C). Caspase-3/7 fluorescence activity assay detected significantly increased activity of caspase-3/7 in ABTB2-OE cells but reduced activity in ABTB2-KO cells (Figure 7D). Together, these results further confirmed that ABTB2 significantly suppresses PDAC, associated with the regulation of cell growth and apoptosis.

### ABTB2 interacts with TRAP1, leading to its degradation through ubiquitination

**Figure 8:**
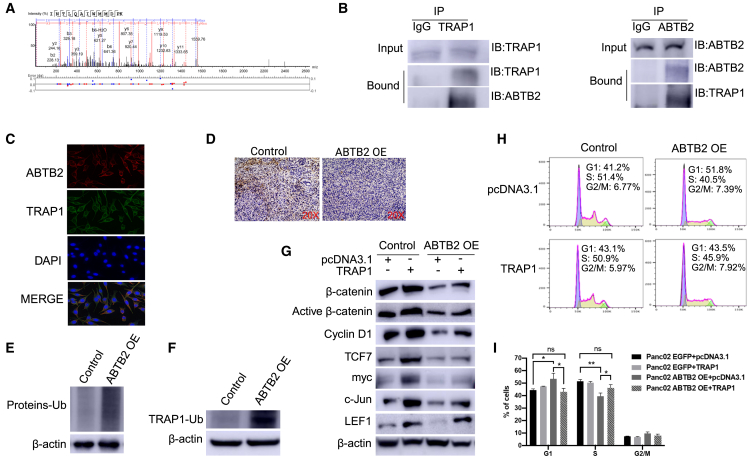
Interaction of ABTB2 and TRAP1 (A) Mass spectrometric spectra showing TRAP1 predicted to interact with ABTB2 by mass spectrometry (B) Co-immunoprecipitation (co-IP) with antibodies against ABTB2 and TRAP1. ABTB2 and TRAP1 alternately served as prey or bait for co-IP, followed by western blotting. The results confirmed that ABTB2 and TRAP1 can be co-precipitated. (C) Immunofluorescence showing intracellular colocalization of ABTB2-TRAP1 in Panc02. Under an immunofluorescence microscope, co-localized ABTB2 and TRAP1 were observed, each labeled with distinct fluorescence markers. The nuclei were stained with DAPI. (D) IHC showing the correlation between ABTB2 and TRAP1. IHC revealed reduced production of TRAP1 in orthotopic pancreatic tumors induced by ABTB2-OE cells. (E and F) Western blotting showing the role of ABTB2 in protein ubiquitination. Notably, western blotting detected markedly enhanced ubiquitination of pan-proteins (E) and TRAP1 (F) in cell lysates from Panc02 cells. Actin was used as a loading control. (G) Western blotting revealed TRAP1 as ABTB2 mediator. Western blotting showed that TRAP1 expression significantly mitigates the effects of ABTB2 on the production of nuclear TCF7, LEF1, c-Myc, c-Jun, and downstream expression of cyclinD1. (H and I) Flow cytometry showing that TRAP1 modulates the effect of ABTB2 on cell cycle regulation. Cell cycle activity was evaluated in ABTB2-OE Panc02 cells with or without TRAP1 plasmid transduction. After staining with propidium iodide, the cells and their respective controls were assessed for their distribution across distinct nuclear phases using a flow cytometer. The results are represented as bar plots (I).

ABTB2, containing a BTB/POZ domain, serves as a crucial hub for protein-protein interactions, orchestrating various cellular processes.^31^ Using immunoprecipitation with anti-ABTB2 Abs, we isolated the ABTB2 complex in ABTB2-OE cells. Characterization of this complex with a mass spectrometer identified TRAP1 as the most potent binding partner of ABTB2 (Figure 8A). Co-immunoprecipitation using Abs specific to ABTB2 and TRAP1, followed by western blotting, confirmed the specific interaction between ABTB2 and TRAP1 (Figure 8B). Immunofluorescence analysis revealed the intracellular colocalization of ABTB2 and TRAP1 (Figure 8C), further validating their interaction. IHC showed reduced *TRAP1* expression in ABTB2-OE cells (Figure 8D), suggesting a negative correlation between ABTB2 and TRAP1. Using western blotting, we detected abundant ubiquitination of whole proteins (Figure 8E) and multiple mono-ubiquitinated TRAP1 (Figures 8F and S10) in ABTB2-OE cells, suggesting that ABTB2-induced protein ubiquitination contributes to TRAP1 degradation. This aligns with earlier research, where investigators demonstrated that the interplay between ABTB2 and Culin 3 facilitates the transfer of ubiquitin to specific substrate proteins for subsequent degradation.^31^ To explore whether TRAP1 contributes to the ABTB2-mediated effect on PDAC cells, TRAP1-expressing plasmids were transduced into ABTB2-OE cells. Western blotting detected that TRAP1 OE counteracted the ABTB2-induced suppression of β-catenin, active β-catenin, cyclin D1, TCF7, Myc, C-Jun, and LEF1 expression (Figure 8G). Additionally, TRAP1 released the ABTB2-induced cell-cycle arrest in the G1 phage (Figures 8H and 8I). These results suggest that ABTB2 interacts with TRAP1, promoting its degradation through ubiquitination, which in turn impacts ABTB2-mediated cellular function and cancer progression.

## Discussion

Leveraging groundbreaking approaches and clinically relevant animal models, we have successfully identified ABTB2 as a previously overlooked yet highly potent suppressive factor in PDAC. This discovery positions ABTB2 as an invaluable target for the development of targeted, effective, and clinically feasible therapeutic interventions. Additionally, detailed mechanistic insights into ABTB2’s signaling pathways and its interaction partners provide a deeper understanding of its critical role in the mechanisms driving PDAC progression, potentially opening new avenues for innovative treatment strategies.

We first identified ABTB2 as an endogenous and previously unrecognized BTB/POZ domain-containing protein with a potent suppressive effect on PDAC. Proteins in this family are involved in various cellular functions, including gene regulation, protein degradation, and cell signaling.^31^^,^^33^^,^^35^^,^^36^^,^^37^^,^^60^ However, only two papers have investigated ABTB2’s role in cancers. In 2001, one study reported that ABTB2 suppresses ovarian tumor cells via the PTEN signaling pathway.^32^ Another study revealed that the upregulation of ABTB2 significantly reduces the apoptosis rate of breast cancer cells, enhancing their resistance to epirubicin.^37^ Employing functional genomic strategies, including CRISPR/cas9, siRNA, shRNA, and expression plasmid, we successfully induced transient and stable KO, KD, or OE of ABTB2 in several human and mouse PDAC cells including mouse Panc02 (Figure 1), UN-KPC-961 (Figure S2), KRAS^G12D^ cells (Figure 6), and human Panc-1 cells (Figure S2). Using these cells and the derived orthotopic models, our comprehensive experiments demonstrated that ABTB2 function, either through gain or loss, profoundly impacts key PDAC cellular processes, including cell proliferation, migration, and apoptosis (Figures 1 and S2) as well as *in vivo* tumor development (Figures 2 and S3). Given the gene mutation of Smad 4 in Panc02 cells, Kras in Kras^G12D^ cells, both Kras and TP53 in UN-KPC-961 and human Panc-1 cells, our findings suggests that ABTB2 may retain its tumor-suppressive potential in the broad context of Kras, TP53, or SMAD4 mutation. Notably, the AKPPC model demonstrated significantly reduced tumor growth and extended survival, underscoring the tumor-suppressive role of ABTB2 (Figure S4). Our consistent findings establish ABTB2 as a potent PDAC suppressive factor in PDAC, providing clear evidence of its potential role in inhibiting tumor progression.

We successfully demonstrated ABTB2 as a potential therapeutic target for PDAC treatment by employing two advanced delivery systems, AAV and LNP. AAV vectors are currently in clinical trials for various cancers but have not yet received Food and Drug Administration (FDA) approval. However, they offer several advantages, including the ability to efficiently deliver genetic material to target cells, a low risk of immune response, and long-lasting expression in both dividing and nondividing cells.^61^^,^^62^^,^^63^^,^^64^ Our pioneering exploration using AAV2-ABTB2 recombinants under the control of the tumor-specific promoter COX2 demonstrated its strong capacity for gene delivery and effective control of both mouse and human PDAC (Figure 7). As an FDA-approved delivery vehicle for mRNA, LNPs have been successfully used in the delivery of mRNA vaccines due to their ability to effectively encapsulate and protect mRNA, enhance cellular uptake, and facilitate efficient release of mRNA into target cells.^65^ Their biocompatibility, low immunogenicity, and ability to target specific tissues make them an ideal delivery system for mRNA and have contributed to their widespread use in mRNA vaccine development.^66^ The safety and efficacy demonstrated in these vaccines have paved the way for the future development of LNP-based mRNA delivery approaches; however, the use of LNPs as a vehicle to deliver mRNA for cancer treatment has not yet been approved.^67^ We pioneered the development of nano-formulated ABTB2 mRNA, named LNP-mRNA-ABTB2, and demonstrated its strong therapeutic capacity in suppressing PDAC; importantly, its combination with the FDA-approved chemotherapeutic agent 5-FU induced a synergistic therapeutic effect (Figure 5). These convincing therapeutic outcomes highlight that the development of ABTB2-targeted interventions using AAV and LNP as delivery systems represents an efficient therapeutic strategy for treating this deadly disease. To this end, we are developing two new delivery systems. Instead of AAV2, AAV8^68^^,^^69^—with high affinity for PDAC—will be selected to deliver and express ABTB2 under the control of the tumor-specific COX2 promoter.^68^^,^^70^^,^^71^ Additionally, we will conjugate LNP with a PDAC-high-affinity peptide to ensure tumor-targeted delivery of ABTB2 mRNA. The dual-control systems, incorporating PDAC-high affinity vehicles and the tumor-specific COX2 promoter, are expected to significantly reduce off-target effects and further enhance therapeutic efficacy, facilitating the advancement of ABTB2-targeted therapies toward clinical application.

We were the first to identify TRAP1 as a key ABTB2-interacting partner, revealing its potential role in cellular processes through this association. As a BTB/POZ domain-containing protein that exerts various cellular functions via BTB- and POZ-domain-mediated protein-protein interactions, we employed immunoprecipitation coupled with mass spectrometry to uncover TRAP1 as its binding partner. ABTB2 promotes TRAP1 degradation via ubiquitination (Figure 8). We also found that ABTB2 interacts with cullin-3 (Figure S9), an E3 ligase component. Together, these results suggest that ABTB2, like other BTB domain-containing proteins, acts as a substrate-specific adaptor to mediate ubiquitin-dependent degradation of TRAP1.^31^^,^^72^^,^^73^^,^^74^ Current studies suggest that TRAP1 ubiquitination-induced subsequent degradation can lead to mitochondrial dysfunction, which in turn induces cellular stress and may trigger cell death, particularly in cancer cells that are highly dependent on mitochondrial function for survival.^75^^,^^76^^,^^77^^,^^78^^,^^79^ Further, TRAP1 degradation significantly reduces cancer cell’s ability to evade apoptosis associated with the regulation of the PI3K/Akt pathway, making them more susceptible to stress-induced cell death.^80^ Clinically, TRAP1 has emerged as a promising therapeutic target,^41^ and several independent groups have developed its inhibitors to evaluate their therapeutic effects in preclinical and clinical trials (NCT04827810). These findings underscore the therapeutic potential of the ABTB2/TRAP1 axis and position ABTB2 as a promising molecular target for future cancer treatment.

In summary, our comprehensive functional, mechanistic, and therapeutic studies have identified ABTB2 as a promising and potent PDAC suppressor, highlighting its potential as a therapeutic target for the development of novel treatment strategies. Furthermore, the discovery of TRAP1 as an interacting partner of ABTB2, along with the elucidation of the underlying molecular mechanisms (Figure S8), provides foundational insights that could serve as a cornerstone for future research and pave the way for innovative clinical applications in PDAC therapy.

## Materials and methods

### Cell culture

Mouse PDAC cell lines UN-KPC-961 (RRID: CVCL_1U13) and Panc02 (RRID: CVCL_D627) were obtained from the National Institute of Health (NIH, Maryland, USA). The mouse Kras^G12D^ cell line, also called UN-KC-6141 (RRID: CVCL_1U11), is derived from the pancreatic tumor of a Kras^G12D^;Pdx1-Cre (KC) mouse at 50 weeks of age and was a gift from Dr. Surinder K. Batra at the University of Nebraska Medical Center.^81^ Human PDAC cell lines Panc-1 (RRID:CVCL_0480) and Mia-paca-2 (RRID:CVCL_0428) were obtained from the American Type Culture Collection (ATCC, Manassas, VA, USA). Cells were selected for this study because they were derived from PDAC tumor-bearing mice or human patients with PDAC. Thus, these cells harbor mutations that closely mimic clinical scenarios and therefore represent valuable cell models for the current study. All cells were confirmed to be free from contamination and cultured at 37°C and 5% CO_2_ in Dulbecco’s Modified Eagle’s Medium (DMEM) (Gibco, NY, USA) supplemented with 10% fetal bovine serum (Gibco), 2 mmol/L L-glutamine, 10 mmol/L HEPES, 100 U/mL penicillin, and 100 μg/mL streptomycin.

### Preparation of recombinant lentivirus with ABTB2 OE, KO, or KD and establishment of stable cell lines

To prepare recombinant lentivirus expressing ABTB2, the lentivirus vector expressing ABTB2 gene was co-transfected into HEK293T cells with two helper plasmids, a packaging plasmid expressing the viral core assembly protein and a plasmid expressing the envelop protein. Twenty-four hours later, the packaged recombinant lentivirus was harvested from the supernatant and used to transduce pancreatic cancer cell lines in the presence of 5 μg/mL polybrene. Eight hours post-transfection, the medium was replaced with normal culture medium supplemented with 500 μg/mL G418 (Mirus Bio LLC, Madison, WI, USA). Two days later, the cells were passaged at low frequency using a limiting dilution method to generate single-cell clones.^82^ Each clone was amplified, and the level of ABTB2 mRNA expression was measured by PCR and western blotting. Similarly, ABTB2 KO or KD lentiviruses were prepared, in which plasmids encoding gRNAs and cas9 or shRNA were used instead of ABTB2-expressing plasmids. These two types of lentiviruses were then used to establish stable cells with ABTB2 KO or KD, as described above.

### Immunohistochemistry staining

As described previously,^82^ pancreas or tumor tissues were fixed with 10% neutral buffered formalin and embedded in paraffin. Tissue sections were processed to conduct IHC. Briefly, tissue sections were de-paraffinized with xylene, rehydrated with various grades of ethanol (100%, 95%, 80%, and 70%), unmasked for antigen retrieval with the provided solution (Vector Laboratories Inc., Burlingame, CA), permeabilized with 0.2% Triton X-100, blocked with serum, and then incubated with BLOXALL reagent (Vector Laboratories Inc., Burlingame, CA) to quench endogenous peroxidase. Subsequently, the sections were incubated successively with primary antibodies, secondary antibodies, and 3,3′-Diaminobenzidine (DAB) substrate at the optimized concentration to develop color. The positive cells were counted in five randomly selected fields on each slide using ImageJ software (National Institutes of Health, Bethesda, MD). Antibodies for ABTB2 (CAT#501734299) purchased from Proteintech (Rosemont, USA), Ki67 (CAT#ab16667), cleaved caspase 3 Ab (CAT#9964S), Bcl-2 (CAT#182858), and β-catenin (CAT#9562L) purchased from Cell Signaling Technology (Danvers, USA) were respectively used to detect expression in tissue sections (Table S2).

### Real-time quantitative PCR

As described previously,^82^ total RNA was extracted from cells using TRIzol reagent (Millipore Sigma, Burlington, MA, USA). Tissues were homogenized using a tissue homogenizer (Fisherbrand 150 Handheld Homogenizer Motor). Reverse transcription of RNA to cDNA was performed using the High-Capacity cDNA Reverse Transcription kit (Applied Biosystems, Foster, CA). qPCR was performed with the QuantStudio 3 Detection System (Thermo Fisher Scientific, Waltham, MA) in a 20 μL reaction mixture containing SYBR Green I (Applied Biosystems, Foster City, CA). The expression levels of different genes were standardized to that of the housekeeping gene 18S rRNA and further analyzed using the 2^−ΔΔCT^ method. All primers were synthesized by Thermo Fisher Scientific (Skokie, IL) and their sequences are shown in Table S1.

### Western blotting

Cells grown on culture plates were rinsed with ice-cold PBS and then lysed in cell lysis buffer (Pierce Biotechnology, Rockford, IL) containing a protease inhibitor mixture (Pierce, CAT#A32959, Rockford, IL, USA). T-PER Tissue Protein Extraction Reagent was added to approximately 10 mg of tissue and homogenized using an electric homogenizer (Fisherbrand 150 Handheld Homogenizer Motor). The lysate was kept on ice for 30 min and centrifuged at 12,000 × g for 10 min at 4°C. The supernatant was boiled with 4× Laemmli sample buffer (BIO-RAD CAT#1610747) containing 5% 2-mercaptoethanol (Gibco, CAT#21985-023, Grand Island, USA) and loaded onto an SDS-polyacrylamide gel. The gel was transferred onto an iBlot polyvinylidene difluoride (PVDF) membrane (Invitrogen, Thermo Fisher Scientific). PVDF membranes were incubated overnight with primary antibodies (see Table S2 for the list of antibodies) at different recommended dilutions. The membranes were then incubated with goat or horse anti-rabbit/mouse IgG at room temperature for 1 h. Finally, protein bands were detected using SuperSignal West Pico PLUS Chemiluminescent Substrate (Thermo Fisher Scientific, Rockford, IL, USA) and imaged using the Amersham Imager 600 (GE Healthcare Bio-Sciences AB, Sweden).

### Colony formation assay

Cells grown to 90% confluence were harvested and seeded in a 6-well plate for culture at a dose of 200 cells per well. 7 to 10 days later, the cells were rinsed with PBS, fixed with glutaraldehyde (6.0% v/v), and stained with crystal violet (0.5% w/v)^83^ for photography and colony counting, as described previously.^82^

### Wound healing assay

Cells grown to 90% confluence were harvested and seed into a 24-well plate with an insert at a density of 2 × 10^5^ cells per well (Wound Healing Assay Kit, Cat#ab242285, Abcam, Cambridge, MA). The insert was carefully removed on the second day. The cell-free gaps were imaged and measured over time using an optical microscope and ImageJ software (NIH).^82^^,^^84^

### Mice

Male C57BL/6 mice, aged six to eight weeks, were purchased from Jackson Laboratory (Bar Harbor, ME). All mouse experiments were performed in accordance with protocols approved by the Institutional Animal Care and Use Committee (IACUC) at the University of Missouri and the University of Connecticut Health Center. All mice received humane care following the criteria outlined in the “Guide for the Care and Use of Laboratory Animals.”

### Orthotopic PDAC model development and survival analysis

Using our established protocol,^84^ different human or mouse PDAC cells grown to 90% confluence were harvested and suspended in PBS containing 15% Matrigel. Subsequently, these cells were implanted into the pancreatic tail of NSG or wild-type C57BL/6 mice (aged 6 to 8 weeks; *n* = 10 per group) at varying doses, depending on the cell line used. For human Panc-1 cells, each NSG mouse received 1 × 10^5^ cells transfected with ABTB2 siRNA; the mice were sacrificed on day 50. Panc-1 cells transfected with scramble siRNA transfection served as controls. For stable Panc02 or UN-KPC-961 cells overexpressing ABTB2-OE, each mouse received 5 × 10^4^ cells and was euthanized on day 35. For stable Panc02 or UN-KPC-961 cells with ABTB2 KO (ABTB2 KO), each mouse received 2.5 × 10^4^ cells and was euthanized on day 30. Stable cells carrying the vector lentivirus were used as respective controls. Tumors harvested from each mouse were weighted and processed for subsequent analyses.

For lifespan analysis, a total BAR (Bright/Alert/Responsive) score of ≥1.0 was defined as the experimental endpoint. At the endpoint, mice were euthanized by exposure to CO_2_ at a flow rate of 3 L/min, followed by cervical dislocation. Kaplan-Meier survival curves were constructed using GraphPad Prism software.^82^ Statistical significance was determined by single-factor analysis of variance and validated using the log-rank test. *p* values of <0.05 were considered statistically significant.

### PDX model

The PDAC tumors from two patients with pancreatic cancer, MPC02 and MPC25, were used to generate PDX model. Briefly, fresh human PDA tumors received from the clinic were digested using collagenase D into a single-cell suspension and implanted into NSG mice via subcutaneous injection at a dose of 0.5 × or 1 × 10^6^ per mouse. The grown tumors were harvested and transplanted into the next cohort of NSG mice. Fourth-generation PDX mice harboring human patient MPC02 tumors, referred to as 4PDX-MPCO2, and fifth generation PDX mice with tumors derived from patient MPC25, referred to as 5PDX-MPC25, were used in this study.

### Construction of AAV2-ABTB2

To prepare the ABTB2-recombinant virus using AAV2 as a vector, mouse Abtb2 cDNA was sub-cloned into the pFB-AAV shuttle plasmid under the control of the human COX2 promoter to generate bacmid DNA (rBV-hCOX2-mAbtb2). This was co-transduced with rBV-inCap2-inRep-hr2 (V104) into insect Sf9 cells to produce the ABTB2-recombinant AAV2 virus (AAV2-ABTB2). After two rounds of purification by CsCl ultracentrifugation, the viral concentration was titrated using qPCR and adjusted to a final concentration of 2 × 10^13^ vg/ml.

### AAV2-ABTB2 treatment

To test the therapeutic efficacy of AAV2-ABTB2 in orthotopic PDAC, an orthotopic mice model was established with Panc02 cells via pancreas inoculation, as described above. On day 11 post-cell inoculation, 100 μL of AAV-ABTB2 (1 × 10^12^ vg/ml) was administered via retro-orbital i.v. injection. Mice were euthanized on day 35’ tumors were harvested, and blood was collected for further analysis. An AAV vector control or PBS was used as controls. To treat PDX tumors, PDX-bearing mice received intratumor injections of AAV2-ABTB2 on day 8, and tumor volumes were measured using a Husky digital caliper. MPC25 PDX mice were euthanized on day 33, while MPC02 PDX mice were euthanized on day 41 post cell inoculation. Tumors were harvested, weighed, and used for further analysis.

### Preparation of LNP-formulated ABTB2 mRNA

The ABTB2 gene was cloned into ProMab’s mRNA standard expression template vector (Richmond, CA, USA), which was subsequently used for *in vitro* transcription to generate modified ABTB2 mRNAs. These modifications included the incorporation of CleanCapAG, a poly-A tail, and pseudonucleotides to enhance mRNA stability and reduce immunogenicity. The modified ABTB2 mRNA was packaged into LNP through an encapsulation process, which involved combining the aqueous mRNA solution with a lipid mixture containing the ethanol phase of SM-102, DSPC, cholesterol, and DMG-PEG2000 using the PreciGenome Flex M System. The generated ABTB2 mRNA-LNPs were purified and concentrated using Amicon Ultra-15 centrifugal filter units (30–100 kDa). Particle size and polydispersity index were determined using the Anton Paar Litesizer 500 system, and encapsulation efficiency was assessed with the Quanti-it RiboGreen RNA assay kit.

### Capability of LNP-mRNA-ABTB2 in producing ABTB2

To test the ability of LNP-mRNA-ABTB2 to produce ABTB2, KRAS^G12D^ cells were seeded into 6-well culture plates at a density of 5 × 10^5^ cells per well. After an overnight culture, cells were transfected with varying doses of ABTB2-mRNA LNP (ranging from 100 ng to 2 μg). 48 h later, ABTB2 expression was assessed using western blotting. All cell culture experiments were conducted in at least four replicates (*n* = 4). Statistical significance is denoted as ∗*p* < 0.05, ∗∗*p* < 0.01, ∗∗∗*p* < 0.001, and ∗∗∗∗*p* < 0.0001.

### LNP-mRNA-ABTB2 monotherapy and combinational therapy with 5-FU

For LNP-mRNA-ABTB2 treatment, a syngeneic model was established in 8-week-old male C57BL/6 mice. Specifically, 0.5 × 10⁶ KRAS^G12D^ cells suspended in 200 μL PBS containing 15% Matrigel were injected subcutaneously into the left flank of each mice. Mice were randomized into four groups and received intratumoral injections of LNP-mRNA-ABTB2 starting on day 5: group 1: PBS control treatment; group 2: 5 μg LNP-mRNA-ABTB2; group 3: 25mg/Kg 5-FU; and group 4: 5 μg LNP-mRNA-ABTB2 + 25 mg/Kg 5-FU. Treatments were repeated twice at 3-day intervals. Tumor volume was measured at 3–4-day intervals using a Husky digital caliper. On day 22 post-tumor inoculation, tumors were harvested and weighed. The animal experiment was performed in triplicate (*n* = 3). Statistical significance was denoted as ∗*p* < 0.05, ∗∗*p* < 0.01, ∗∗∗*p* < 0.001, and ∗∗∗∗*p* < 0.0001.

### RNA sequencing

Total RNA was exacted from ABTB2-KD KRAS^G12D^ cells using TRIzol reagent, as previously described. Approximately 2 μg of RNA was enriched using oligo (dT) to remove rRNA. RNA-seq libraries were prepared using the KAPA RNA-Seq Library Prep Kit (Roche). Completed libraries were quality-checked with an Agilent 2100 Bioanalyzer and quantified by absolute quantification qPCR. Libraries were barcoded, denatured into single-stranded DNA with NaOH, captured on an Illumina flow cell, amplified *in situ*, and sequenced for 150 cycles on both ends using an Illumina NovaSeq 6000 instrument. Sequence quality was examined using FastQC software. Cutadapt was used to trim the 5′ and 3′ ends, and reads were aligned to the reference genome using Hisat2 software. Transcript abundance for each sample was estimated using StringTie, and FPKM values at both the gene and transcript levels were calculated using the R package Ballgown. Differentially expressed genes (DEGs) and transcripts were identified and filtered using Ballgown. PCA and correlation analyses were performed based on gene expression levels. Hierarchical clustering, GO, and pathway analysis were performed on the DEGs using R, Python, or shell environments for statistical computing and graphics.

### TOP-FLASH assay

Cells were seeded into 96-well plates at a density of 8 × 10^3^ cells per well and transfected with the T-cell factor/lymphoid enhancer factor (TCF/LEF) luciferase reporter vector or a negative control reporter vector from the TCF/LEF Reporter Kit (BPS Bioscience, San Diego, CA), according to the manufacturer’s instructions. 48 h after transfection, cells were harvested and lysed with the passive lysis buffer provided in the Promega Luciferase System (Promega, Madison, WI). Luciferase and Renilla activities were determined using a luminometer (BPS Bioscience, San Diego, CA). Relative luciferase activities were obtained by normalizing luciferase activity to Renilla activity. Data are presented as mean ± SE (*n* = 3).

### Cell cycle analysis

Cells were fixed with 70% ethanol at −20°C overnight and stained with 50 μg/mL PI for 45 min. Cell cycle progression in stained cells was determined using an LSR Fortessa X-20 flow cytometer (BD Bioscience, San Jose, CA). Data were analyzed using FlowJo software (Tree Star; https://www.flowjo.com/).

### Caspase 3/7 activity assay

Cells were seeded into 96-well plate at a density of 8 × 10^3^ per well and grown to 90% confluence. The plates were then incubated with caspase 3/7 reagent (Promega, Madison, WI) for 5 h. Fluorescent readings were recorded using an excitation wavelength of 495 nm and an emission wavelength of 535 nm. For staurosporine-induced apoptosis, cells were cultured with 1 μM of staurosporine or 10 μM of DMSO for 24 h before the addition of caspase-3/7 reagent. The assay was conducted in triplicate, and readings were acquired using a plate reader (spectramax ID3, molecular devices, San Jose, CA).

### Apoptosis assay

Cells grown to 80% confluence were incubated with 1 μM staurosporine (Millipore Sigma) or DMSO for 24 h, then stained using the Apoptosis Detection Kit (Cat#: 556547, BD Bioscience, San Jose, CA). Stained cells were analyzed using an LSR Fortessa X-20 flow cytometer (BD Bioscience, San Jose, CA). Data were analyzed using FlowJo software.

### Immunoprecipitation and mass spectrometry

Following our previous described procedure,^85^ Panc02 cells grown to approximately 80% confluence were rinsed twice with ice-cold PBS and lysed with cold cell lysis buffer (Pierce Biotechnology, Rockford, IL) containing a protease inhibitor mixture (Pierce, CAT#A32959, Rockford, IL, USA). Lysates were diluted 4-fold with cold dilution buffer containing 1 × protease inhibitor, then clarified by centrifugation at 15, 000 rpm for 30 min at 4°C. The resulting supernatant was incubated with Protein A-coated Dynabeads magnetic beads (Invitrogen, Carlsbad, CA) preincubated with anti-ABTB2 antibody (Santa Cruz, Dallas, Texas, USA). After 10 min, the beads were harvested and washed 3 times with wash buffer, followed by elution with mild elution buffer. The protein eluent was dissolved in urea buffer and purified by acetone precipitation, followed by enzymatic digestion and chemical modification through the reduction of disulfide linkages and alkylation of free cysteinyl thiol groups. The modified peptides were concentrated using a C18 ziptip and subjected to mass spectrometry on a Bruker timsTOF Pro spectrometer. The data were analyzed using PEAKS (version X+) software, with Uniprot selected as the reference protein sequence database.

### Statistical analysis

Statistical significance between groups was determined using one-way ANOVA (for ≥3 groups), followed by the recommended correction with Tukey’s multiple comparison test, or using a one-tailed or two-sided Mann-Whitney test (two groups) with GraphPad Prism software (version 8.3.0, GraphPad Software, La Jolla, CA). Data are represented as mean ± standard deviation (SD). A *p* value < 0.05 was considered statistically significant.

### Ethics approval and consent to participate

The present study was performed under protocols approved by the Institutional Review Board (IRB) of Baylor School of Medicine and the Institutional Animal Care and Use Committee (IACUC) at the University of Missouri and the University of Connecticut Health Center. All mice received humane care according to the criteria outlined in the “Guide for the Care and Use of Laboratory Animals.”

## Data availability

All data necessary to evaluate the conclusions in the paper are present in the paper and/or the supplemental information.

## Acknowledgments

This project was supported by grants from a startup fund from the 10.13039/100007165University of Missouri (G.L., PI), a startup fund from the UConn Health Center (G.L., PI), and the Siteman Investment Program (SIP) research development award (PIs: G.L. and David DeNardo).

## Author contributions

Conception and design, N.L., O.T.O., Q. W., Y.M., K.F.S.-O., and G.L.; development of methodology, N.L., O.T.O., Q.W., H.J., E.R.C., K.C., E.T.K., and G.L.; acquisition of data, N.L., O.T.O., Q.W., H.J., X.L., and G.L.; funding acquisition, K.F.S.-O. and G.L; data analysis and interpretation, N.L., O.T.O., H.J., E.R.C., and G.L.; figure preparation, N.L., O.T.O., H.J., and G.L.; writing the manuscript, O.T.O., N.L., K.C., K.F.S-O., and G.L; study supervision, Y.M., K.F.S.-O., and G.L. All authors read and approved the final version of the manuscript.

## Declaration of interests

The authors declare no competing interests.

## Supplemental information

Document S1. Figures S1–S10 and Tables S1 and S2

Document S2. Article plus supplemental information
