# Supplementary material for: Mechanistic and functional characterization of ABTB2 as a novel target for pancreatic cancer therapy
Source: Mol Ther Oncol. 2025 Nov 1;33(4):201080. doi: 10.1016/j.omton.2025.201080 (PMC12664368; doi:10.1016/j.omton.2025.201080)
Supplement: Document S1. Figures S1–S10 and Tables S1 and S2 [file mmc1.pdf]

OMTON, Volume *33*

Supplemental information

Mechanistic and functional characterization of ABTB2 as a novel target for pancreatic cancer therapy

Nan Lyu, Olamide T. Olaoba, Qiongling Wang, Harinarayanan Janakiraman, Xinjian Liu, Ernest Ramsay Camp, Kun Cheng, Eric T. Kimchi, Yi Miao, Kevin F. Staveley-O'Carroll, and Guangfu Li

Supplemental Figures

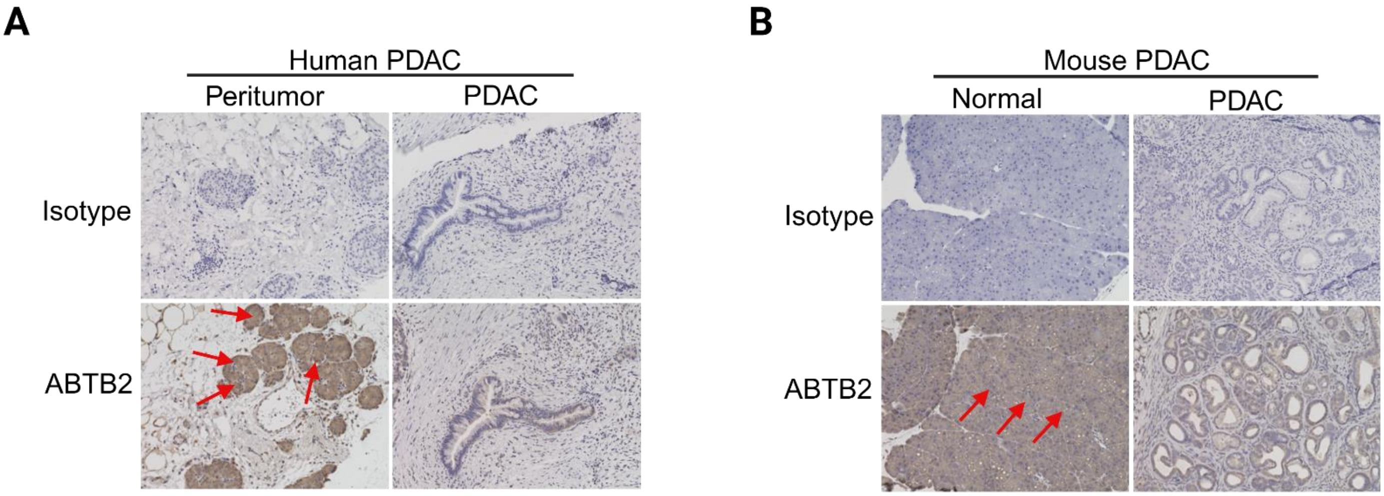

Figure S1: Expression of ABTB2 in mice and human PDAC tumors. (A) The reduced expression of ABTB2 in human PDAC tumors. IHC detected the obviously decreased expression of ABTB2 in human PDAC tumors compared to that in peritumor tissue. **(B) The reduced ABTB2 production in mouse PDAC tumors.** IHC detected the reduced expression of ABTB2 in mouse PDAC tumors from genetically modified KPC mice compared to that in pancreas from normal C57BL/6 mice.

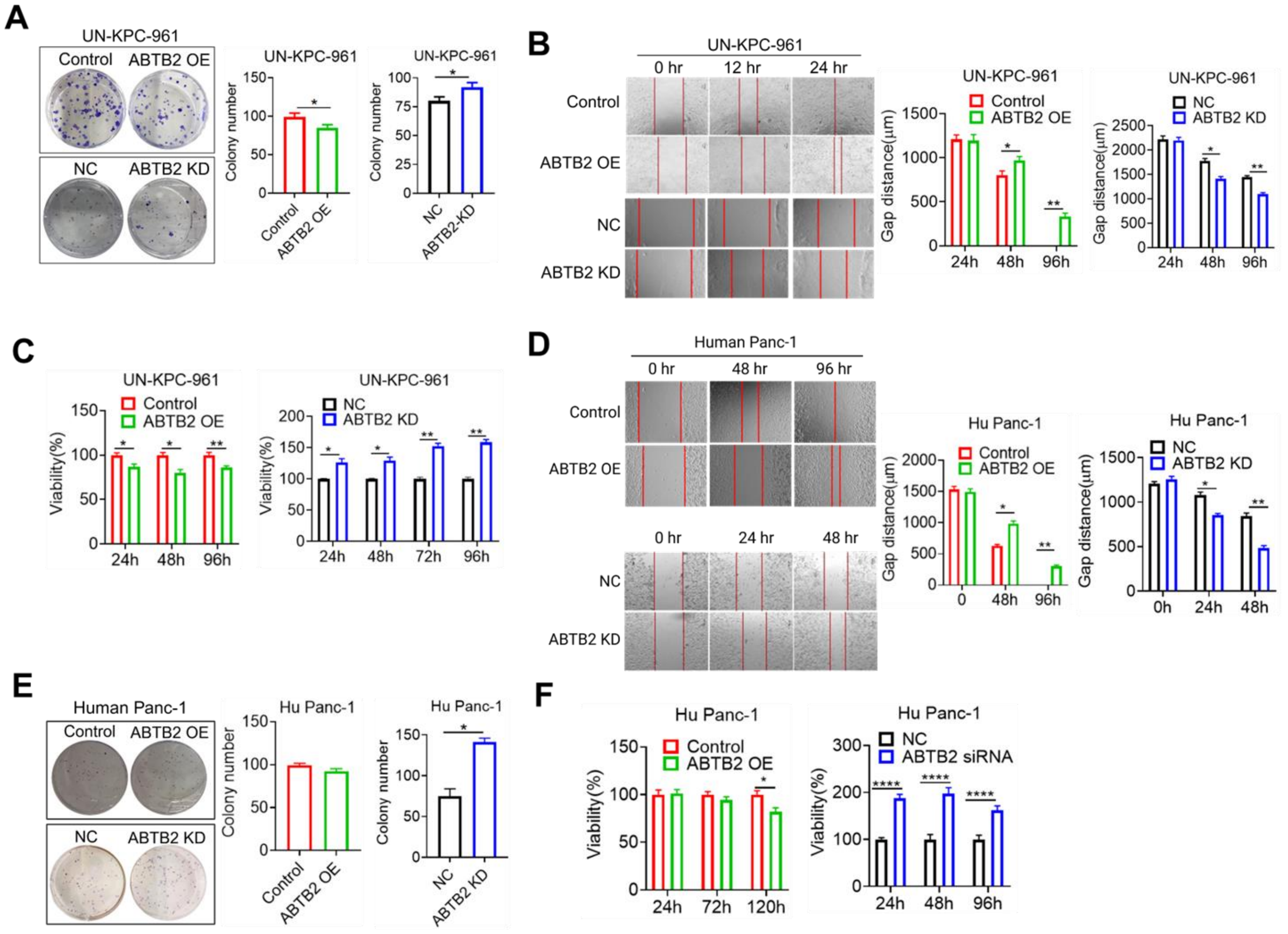

Figure S2: ABTB2 suppresses mouse and human PDAC cell growth. (A) ABTB2-OE or AB TB2-KD on mouse UN-KPC-961 cell colony formation. Mouse UN-KPC-961 cells with ABTB2-OE or ABTB2-KO were seeded into 6-well plates at a low-density of 200 cells/well for culture, 7 to 10 days later, cells were then fixed with 6% v/v glutaraldehyde and stained with 0.5% w/v crystal violet, and the formed cell colonies were imaged and counted. (B) **ABTB2-OE or AB TB2-KD on mouse UN-KPC-961 cell migration.** UN-KPC-961 PDAC cells with ABTB2-OE or ABTB2-KO were seeded into 24-well plate with a wound insert which was removed in the second day. The wound closure was imaged, and cell-free gaps were daily measured. **(C) Impact of ABTB2 KO and OE on UN-KPC-961 cell viability.** UN-KPC-961 cells with ABTB2-OE or ABTB2-KO were seeded into 96-well plate. After 16-hour culture, 10μL of MTT labeling reagent was added to each well and incubated for 3 hours,

then the absorbance of purple formazan crystal was measured. The cell viability with ABTB2 OE (left panel) and ABTB2 KO (right panel) were calculated as a percentage relative to the respective control. (D) **ABTB2-OE or AB TB2-KD on mouse human migration Panc-1 cell migration.** This experiment was conducted as described in (B). (E) **ABTB2-OE or AB TB2-KD on mouse human migration Panc-1 cell colony formation.** This experiment was conducted as described in (A). **(F) Impact of ABTB2 KO and OE on human Panc-1 cell viability.** This experiment was conducted as described in (C). n=3, error bars represent mean ± SD, statistical significance is denoted as *P < 0.05, ** P < 0.01, *** P < 0.001.

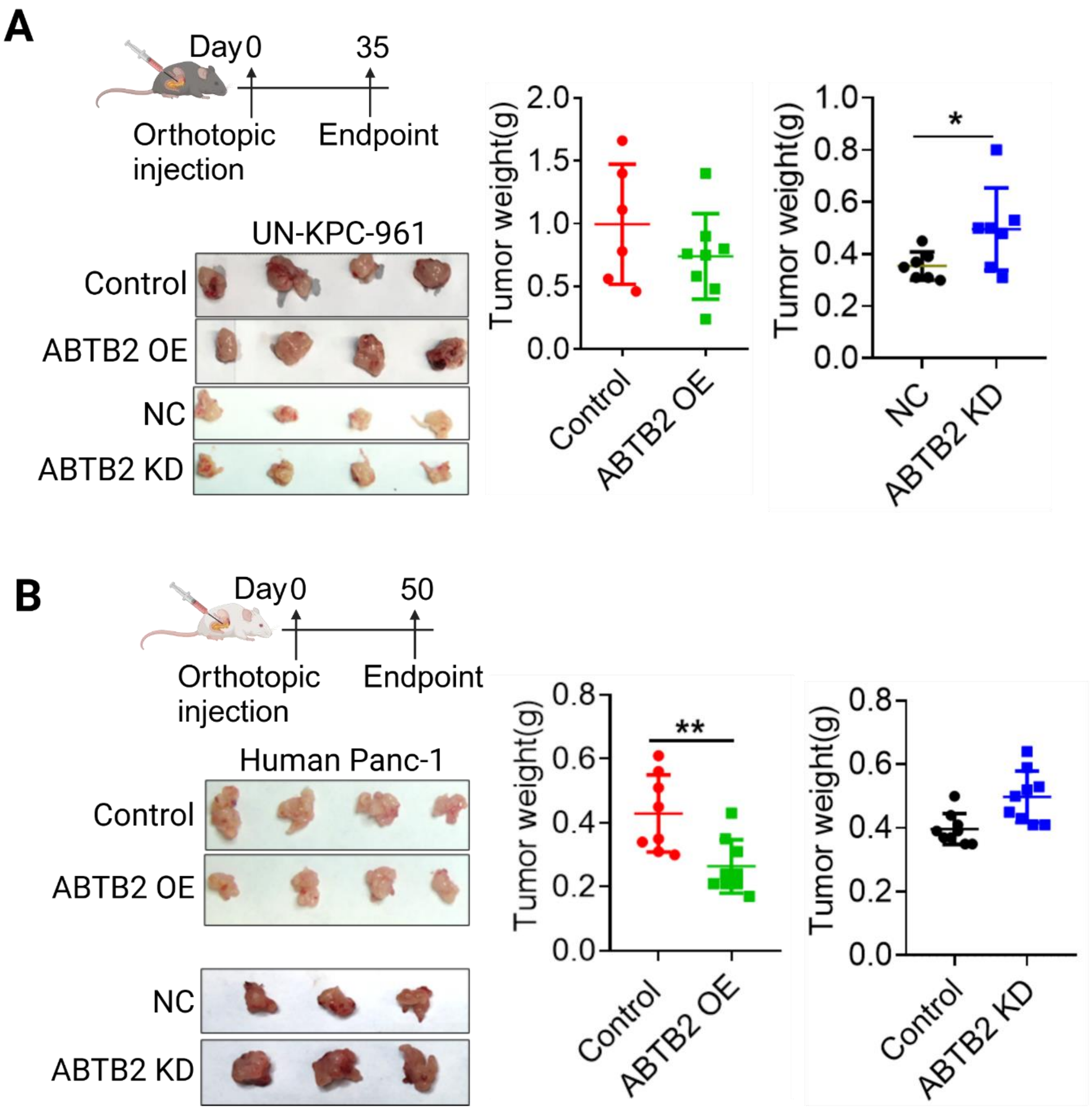

Figure S3: Intrinsic dysregulation of ABTB2 in mouse PDAC cells affects orthotopic tumor growth. (A) The growth of orthotopic PDAC tumors induced by UN-KPC-961 cells with ABTB2-OE or -KD. UN-KPC-961 cells with ABTB2 OE or KD were injected into pancreas of wild type C57BL/6 mice at a dose of 5 x 10^4 and 5 x 10^4 cells per mouse. The corresponding vehicles were used as controls. After 35 days, mice were euthanized, and tumors were harvested for imaging and weighing. **(B) The growth of orthotopic PDAC tumors induced by human Panc-1 cells with ABTB2-OE or -KD.** Panc-1 cells with ABTB2-OE or KD were injected into pancreas

of immunodeficient NSG mice. After 50 days, mice were euthanized, and tumors were harvested for imaging and weighing. n=8, statistical significance is denoted as *$P < 0.05$, ** $P < 0.01$.

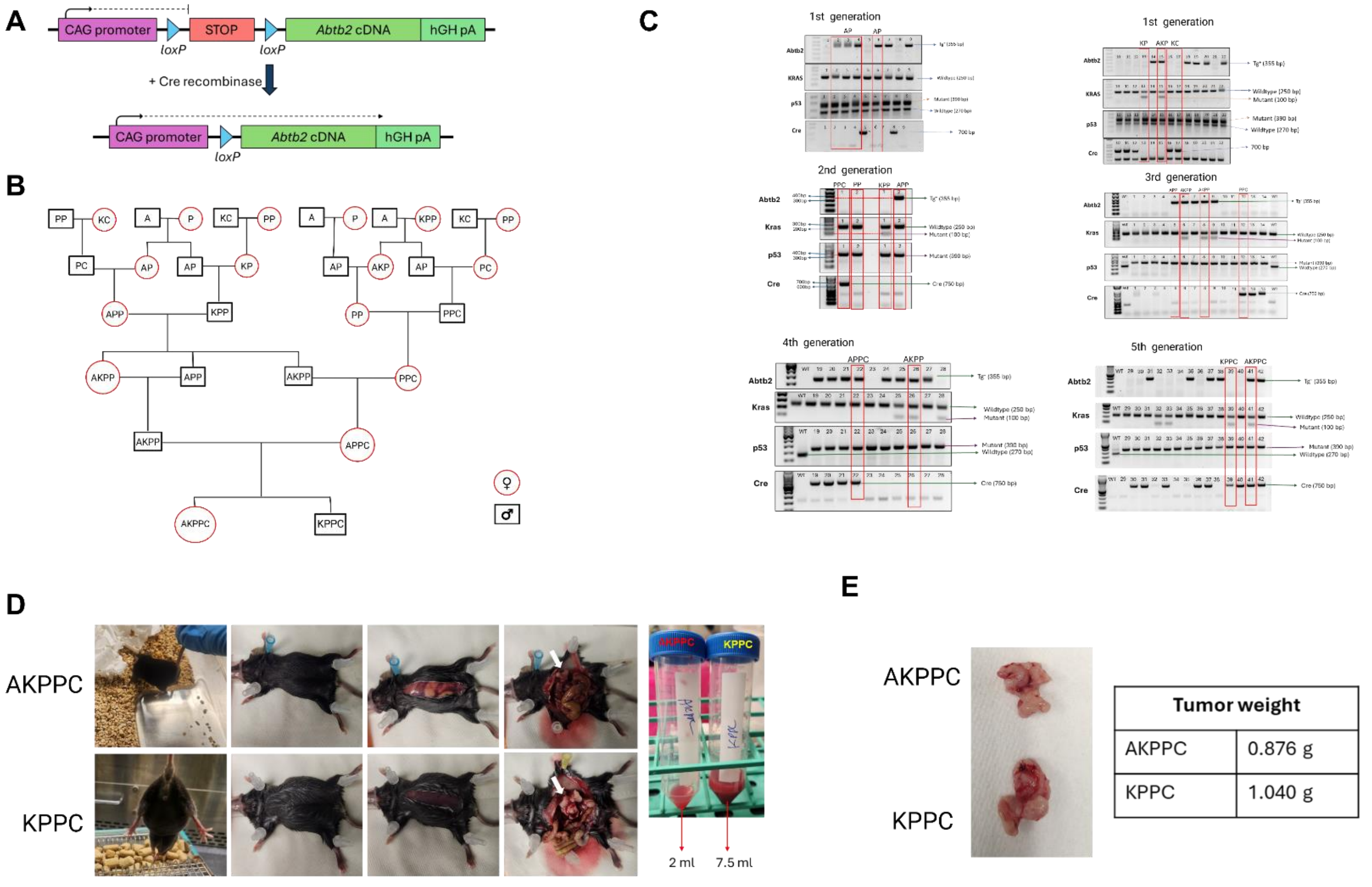

Figure S4: Establishment of AKPPC and KPPC spontaneous PDAC model. (A) Schematic of the ABTB2 transgene construct in a Cre-LoxP system. The ABTB2 expression cassette is driven by the CAG promoter, with a LoxP-flanked stop sequence positioned upstream the ABTB2 cDNA. In the presence of pancreas-specific p48-Cre recombinase, the stop cassette is excised, enabling ABTB2 expression specifically in the pancreas. **(B) Breeding scheme for the generation of AKPPC and KPPC spontaneous PDAC model:** Mice carrying homozygous $Trp53^{R172H}$ mutations (designated as PP) was crossed with $Kras^{G12D}$; p48-Cre transgenic mice (designated as KC). Separately, ABTB2 transgenic mice (A) were crossed with $Trp53^{R172H}$ heterozygous mice (AP). Additional breeding combinations include KC x PP and A x KPP. In the F1 generation, offspring with genotypes PC, AP, KP, and AKP were obtained. These F1 generation were further bred (PC x AP, AP x KP, AP x AKP, and AP x PC) to produce F2 progeny with APP, KPP, PP, and PPC genotypes. Subsequent breeding (APP x KPP and PP x PPC) generated F3 mice with AKPP, APP, and PPC genotypes. These were then crossed (AKPP

x APP and AKPP x PPC) to yield F4 mice AKPP and APPC genotypes. Finally, F4 mice with AKPP and APPC mice were crossed to produce the desired AKPPC and KPPC mice in the 5th generation. **C. Genotyping by agarose gel electrophoresis:** Tail tips were collected at weaning. Genomic DNA was isolated and genotyped using specific primers targeting ABTB2 and p48-Cre, Trp53^{R172H}, and Kras^{G12D}. PCR products were resolved by agarose gel electrophoresis to confirm transgene integration and mutation presence. **D. Phenotypic evaluation of AKPPC and KPPC mice:** Mice were monitored daily starting at 6 weeks of age for signs of disease progression, including hemorrhagic ascites and behavioral changes (BAR score). At 2 months and 3 days, KPPC mice were euthanized due to severe ascites and a BAR score >1. In contrast, AKPPC mice remain asymptomatic and displayed normal activity at the same time point. **E. Tumor burden assessment.** Tumors harvested from AKPPC and KPPC were excised and weighed for comparison.

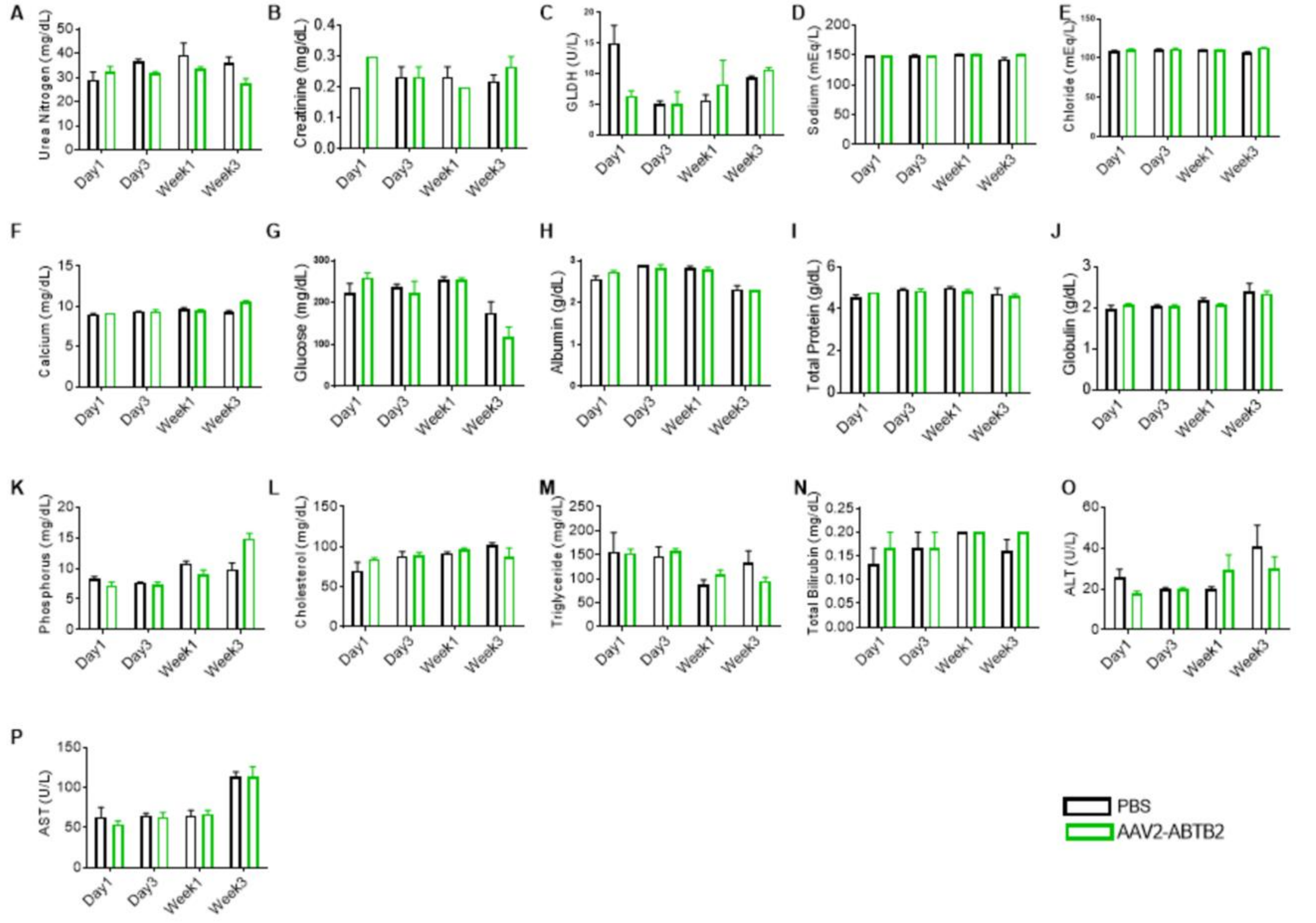

Figure S5: Evaluation of AAV2-ABTB2 toxicity. C57BL/6 mice with orthotopic PDAC tumors induced by Panco2 cells were divided into two groups: AAV2-ABTB2 and control PBS treatment. Two weeks after injection, mice were examined and anesthetized to harvest blood, plasma, and pathologic specimens. Clinical assessment, blood analysis, and pathologic evaluation were carried out in the Veterinary Medical Diagnostic Laboratory at the University of Missouri College of Veterinary Medicine. Renal toxicity was evaluated by assessing markers of glomerular filtration rate and absorption/reabsorption including **(A)** Blood Urea Nitrogen (BUN), **(B)** Creatinine, **(C)** Glutamate dehydrogenase (GLDH). **(D)** Sodium, **(E)** Chloride, and **(F)** Calcium were measured. Hepatotoxicity was evaluated by measuring the following parameters including **(G)** Glucose, **(H)** Albumin, **(I)** Total Protein, **(J)** Globulin, **(K)** Phosphorus, **(L)** Cholesterol, **(M)** Triglyceride, **(N)**Total Bilirubin, **(O)** Alanine Aminotransferase (ALT), and **(P)** Aspartate transaminase (AST)

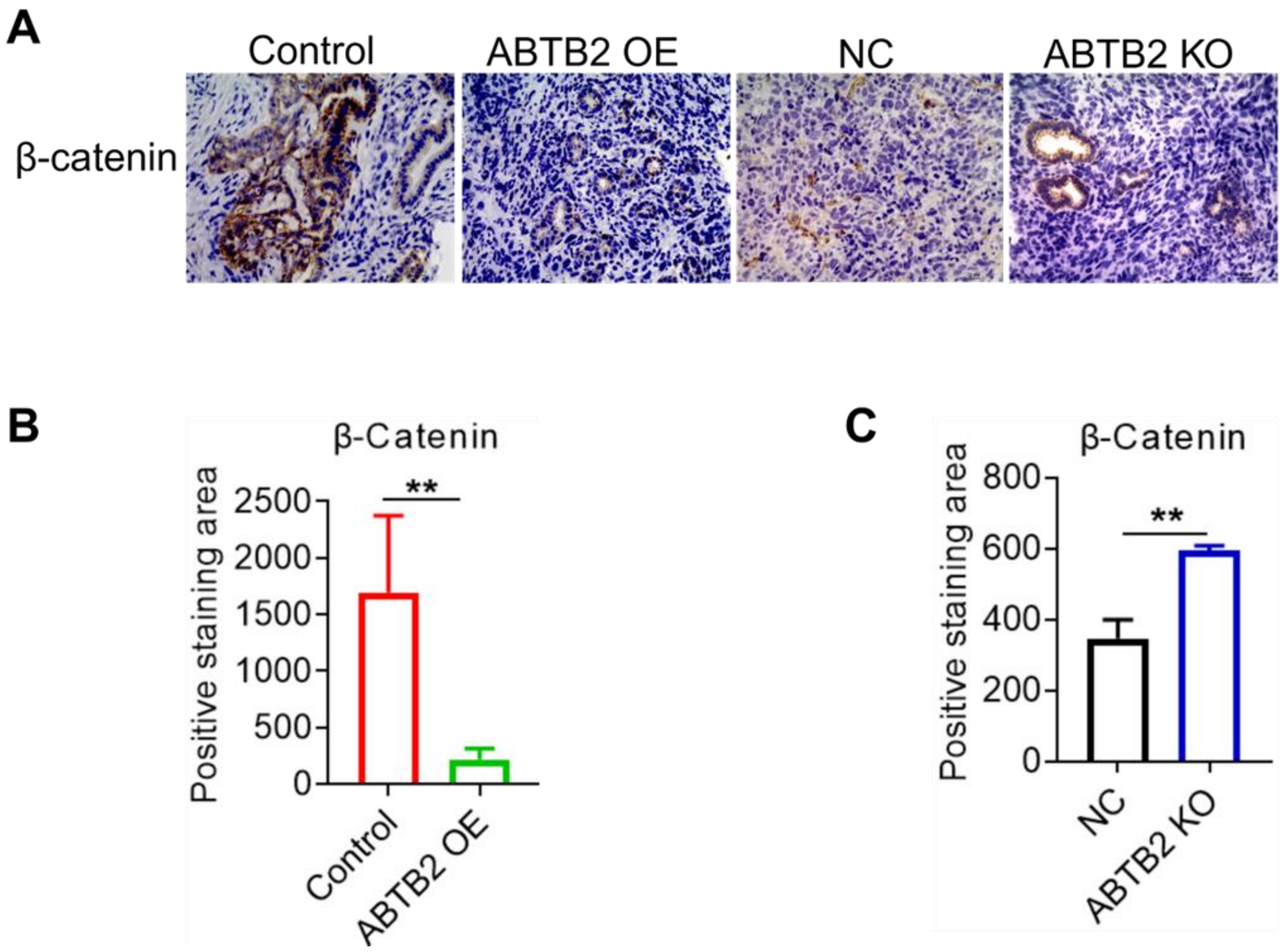

Figure S6: ABTB2 dysregulation affects β-catenin expression. (A) Representative images of IHC staining. IHC staining showed the reduced expression of β-catenin in orthotopic PDAC tumors induced by Panc02 cells with ABTB2 OE, vice versa the increased expression in PDAC tumors induced by Panco2 cells with ABTB2 KO. **(B & C)** Semi-quantitative measurement of positive staining area of β-catenin in figure (A).

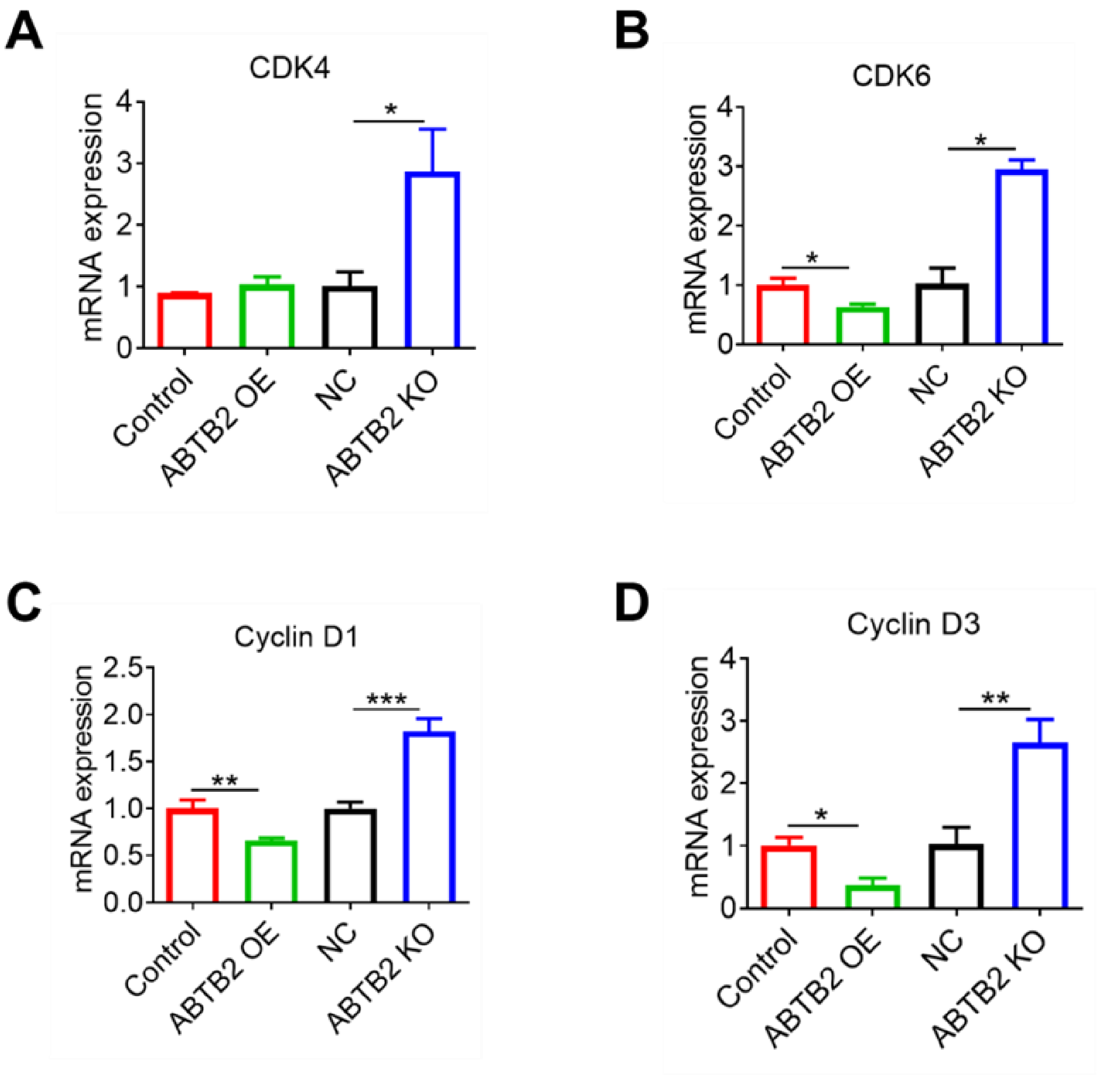

Figure S7: Dysregulation of ABTB2 modulates the expression of critical cell cycle genes. qPCR assay indicated that ABTB2 OE or KO affects Panc02 mRNA expression of CDK 4 **(A)**, CDK6 **(B)**, cyclin D1 **(C)**, and cyclin D3 **(D)**.

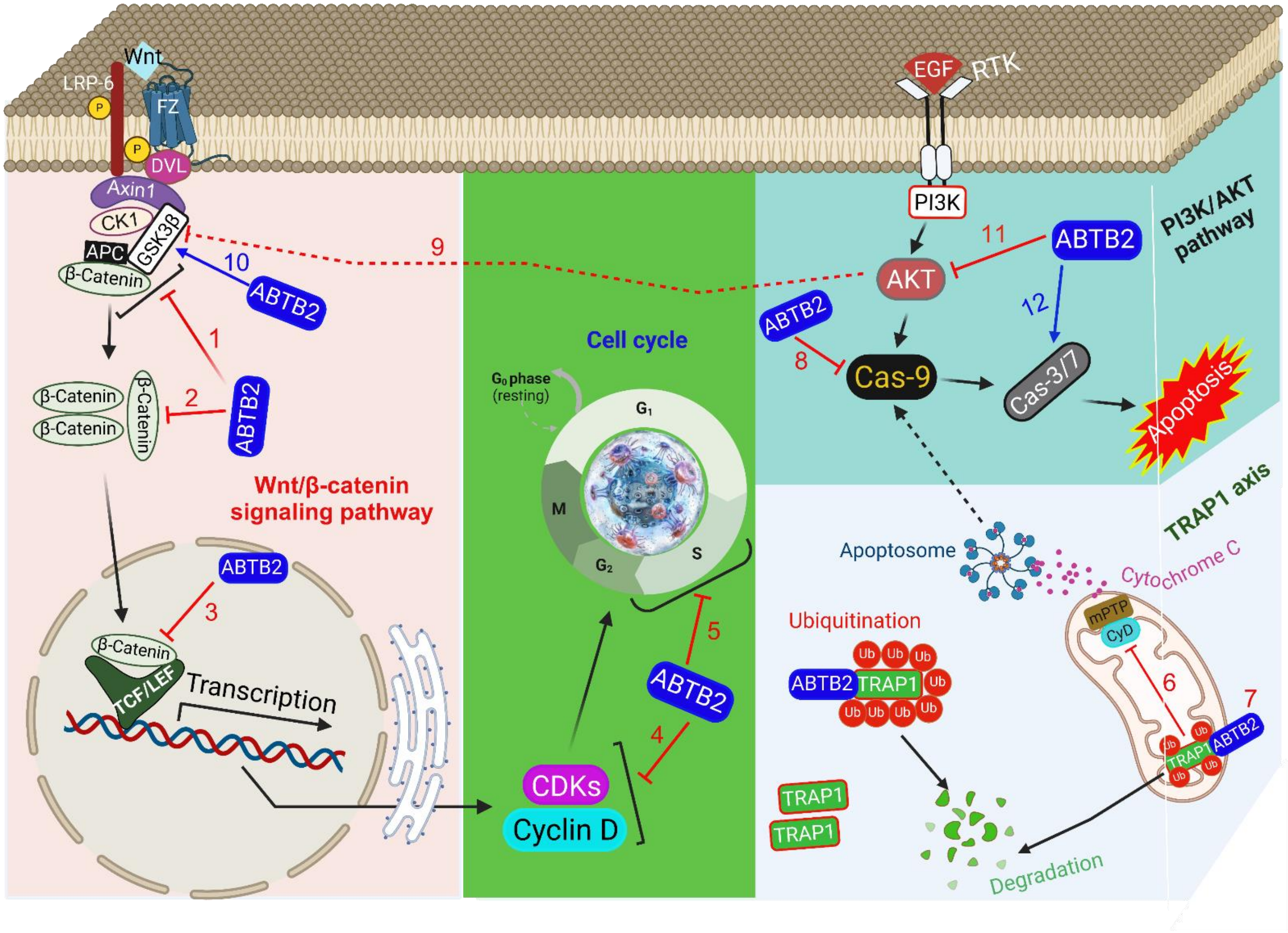

Figure S8: Mechanisms of ABTB2-mediated growth suppression in PDAC cells. ABTB2 modulates PDAC cellular processes and behavior by playing multifaceted functions, which is involved in regulation of Wnt/β-catenin pathway to suppressing cell proliferation and survival by suppressing β-catenin, TCF/LEF, and MyC; cell cycle signaling to modulate cell cycle progression by decreasing the expression of cyclin D1/D3, CDK4, CDK6, and Ki-67; and apoptosis signaling to trigger caspase activation by downregulating Bcl-2, Survivin, and upregulating Caspase 3/9 and cleaved PARP. In addition, ABTB2 interacts with TRAP1, leading to its degradation through ubiquitination, which may contribute to β-catenin reduction and impaired mitochondrial function.

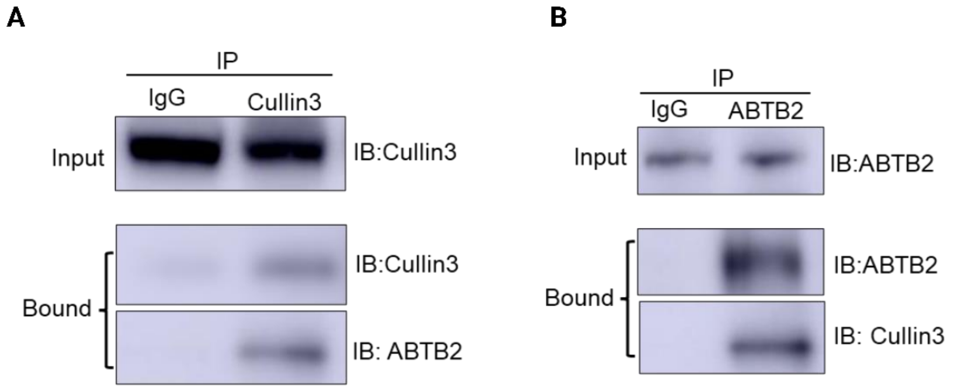

Figure S9: ABTB2 interacts with cullin-3 protein as an adaptor. (A-B). CO-IP experiment was performed to confirm the interaction of cullin-3 and ABTB2. ABTB2 was detected in the pull-down fraction of Cullin3 and vice versa.

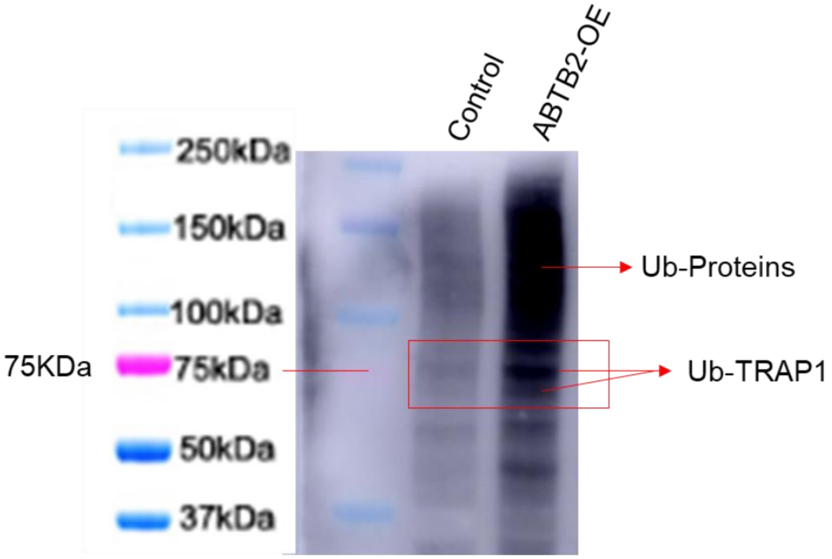

Figure S10: Western blotting showing multiple mono-ubiquitination of TRAP1. Cell lysate of ABTB2-OE Panc02 cells were incubated with Pan-Ub antibody. Cell lysate from ABTB2 OE showed higher ubiquitination level compared to control, and ubiquitinated TRAP1 increased in ABTB2 OE cells

Supplemental Tables

Table S1: List of primers

mus-Abtb2-F	ACACTGGAGCAGTCACTCCT
mus-Abtb2-R	TGGCTCGAGAGACCAGATCA
human-ABTB2-F	TGCGGCAAGAACGCCAATG
Human-ABTB2-R	ACGGGAGACCAAGTCACTCAGCT
mus-CDK4-F	GTCTATGGTCTGGCCCGAAG
mus-CDK4-R	CGGGTTCATATCGAGTGGCA
mus-CDK6-F	ACCTCTCCTTCGTGAAGACTG
mus-CDK6-R	CCCTCCGCAGCGATTACATAG
mus-CyclinD1-F	TCAAGTGTGACCCGGACTGC
mus-CyclinD1-R	CCTTGGGGTCGACGTTCTG
mus-CyclinD3-F	GGACACTCGCTTTGTTTGGG
mus-CyclinD3-R	AGCATTTCAGGGCGAGCTTA
mus-Ctnnb1-F	TATGGACTGCCTGTTGTGGT
mus-Ctnnb1-R	TCGTGGAATAGCACCCTGTT
Mus-18s-F	AAGTCCCTGCCCTTTGTACACA
Mus-18s-R	GCCTCACTAAACCATCCAATCG
h-18S-F	GTAACCCGTTGAACCCCATT
h-18S-R	CCATCCAATCGGTAGTAGCG

Table S2 List of antibodies

Antibody	Host	Vendor	Catalog #
Met	Rabbit	Cell signaling	8198T
ABTB2	Rabbit	Proteintech	24323-1-AP
CDK4	Rabbit	Cell signaling	12790T
p-Akt	Rabbit	Cell signaling	2965S
c-Jun	Rabbit	Cell signaling	9165T
TCF4/TCF7	Rabbit	Cell signaling	2569T
GAPDH	Rabbit	Cell signaling	8884S
β-actin	Rabbit	Cell signaling	4967S
p-GSK-3β (S9)	Rabbit	Cell signaling	5558T
Cytochrome C	Rabbit	Cell signaling	4272S
c-Myc	Rabbit	Cell signaling	5605T
p-Akt (S473)	Rabbit	Cell signaling	4058S; 4060S
Bcl2	Rabbit	Cell signaling	3498S
Survivin	Rabbit	Cell signaling	2808S
β-catenin (S45)	Rabbit	Cell signaling	19807S
Cyclin D1	Rabbit	Cell signaling	2978S
Akt	Rabbit	Cell signaling	9272S
LEF1	Rabbit	Cell signaling	2230T
TCF1/TCF7	Rabbit	Cell signaling	2203S
GSK-3β	Rabbit	Cell signaling	12456S
p-Rb (S780)	Rabbit	Cell signaling	8180S
p-Rb (807/811)	Rabbit	Cell signaling	8516S
Met	Mouse	Cell signaling	3127L
Axin1	Rabbit	Cell signaling	2087S
p-LRP6	Rabbit	Cell signaling	2568S
Cyclin D3	Mouse	Cell signaling	2936S
Cleaved PARP	Rabbit	Cell signaling	94885S
LRP6	Rabbit	Cell signaling	3395S
Cleaved PARP	Mouse	Cell signaling	9548S
p-β-catenin (S552)	Rabbit	Cell signaling	5651T
p-β-catenin (S675)	Rabbit	Cell signaling	4176T
LRP6	Rabbit	Cell signaling	3395T
p-LRP6	Rabbit	Cell signaling	2568T
pGSK-3β (S21/9)	Rabbit	Cell signaling	8566T
p-Akt (7500)	Rabbit	Cell signaling	2965S
Ki67	Rabbit	Abcam	16667
Bad	Rabbit	Cell signaling	9239S
CD31	Rabbit	Abcam	28364
